# NF-κB Signaling and Inflammation—Drug Repurposing to Treat Inflammatory Disorders?

**DOI:** 10.3390/biology11030372

**Published:** 2022-02-26

**Authors:** Annabell Roberti, Laura Elizabeth Chaffey, David R. Greaves

**Affiliations:** Sir William Dunn School of Pathology, University of Oxford, South Parks Road, Oxford OX1 3RE, UK; annabell.roberti@path.ox.ac.uk (A.R.); laura.chaffey@path.ox.ac.uk (L.E.C.)

**Keywords:** inflammation, NF-κB, drug repurposing, drug development, autoimmunity, COVID-19, multiple sclerosis, rheumatoid arthritis

## Abstract

**Simple Summary:**

Since its first description 35 years ago, the transcription factor NF-κB (nuclear factor κ-light-chain-enhancer of activated B cells) has been shown to be a key mediator of immune cell responses to inflammatory mediators, oxidative stress and genotoxic injury. Dysregulated NF-κB signalling drives inflammation in inflammatory disorders such as multiple sclerosis, rheumatoid arthritis or inflammatory bowel disease. Thus, re-establishing the appropriate regulation of NF-κB activity seems like a promising approach to treat inflammatory diseases. Current anti-inflammatory drugs have many, often serious, side effects. Thus, there is an unmet clinical need for safe and effective anti-inflammatory medicines that both decrease inflammatory mediator production and enhance endogenous anti-inflammatory and prorepair pathways. So far, traditional de novo drug discovery has fallen short of satisfying this need. Drug repurposing is a cost- and time-effective alternative to de novo drug development for the identification of novel applications and has already resulted in the identification of effective anti-inflammatories in the ongoing COVID-19 pandemic. In this paper we critically review NF-κB as a potential target for the development of anti-inflammatory drugs with an emphasis on drug repurposing as a strategy to identify new approaches to treat inflammatory diseases.

**Abstract:**

NF-κB is a central mediator of inflammation, response to DNA damage and oxidative stress. As a result of its central role in so many important cellular processes, NF-κB dysregulation has been implicated in the pathology of important human diseases. NF-κB activation causes inappropriate inflammatory responses in diseases including rheumatoid arthritis (RA) and multiple sclerosis (MS). Thus, modulation of NF-κB signaling is being widely investigated as an approach to treat chronic inflammatory diseases, autoimmunity and cancer. The emergence of COVID-19 in late 2019, the subsequent pandemic and the huge clinical burden of patients with life-threatening SARS-CoV-2 pneumonia led to a massive scramble to repurpose existing medicines to treat lung inflammation in a wide range of healthcare systems. These efforts continue and have proven to be controversial. Drug repurposing strategies are a promising alternative to de novo drug development, as they minimize drug development timelines and reduce the risk of failure due to unexpected side effects. Different experimental approaches have been applied to identify existing medicines which inhibit NF-κB that could be repurposed as anti-inflammatory drugs.

## 1. NF-κB Signaling in Inflammation

### 1.1. A Brief History of NF-κB Signaling in Inflammatory Diseases

The transcription factor NF-κB (nuclear factor κ-light-chain-enhancer of activated B cells) is named for its 1986 discovery in B cells, in which it was found to bind to the enhancer element of the κ-IgG chain gene [1]. In a broader context, NF-κB is expressed in almost all cell types [2] and is involved in essential cellular processes such as apoptosis and cell cycle progression [3]. In immune cells, NF-κB is key in the response of innate cells to viral or bacterial antigens and other stimuli such as cytokines during inflammation [4]. Despite its name, NF-κB signaling is an important regulator of the transcription of genes such as cytokines, chemokines or interferon-stimulated genes (ISGs) in innate immune cells [5]. As a result of its central role in many cellular processes, NF-κB dysregulation has been implicated in the pathology of numerous diseases. In several cancer types, NF-κB is constitutively activated, resulting in unregulated proliferation, thus making it an important therapeutic target in many cancers such as breast cancer, lung cancer, gastric and colorectal cancer as well as hematologic malignancies [2,6,7,8,9,10,11]. As a central mediator of inflammation, NF-κB activity causes inappropriate inflammatory responses in rheumatoid arthritis (RA), inflammatory bowel disease (IBD), multiple sclerosis (MS) and atherosclerosis [12,13]. Thus, modulation of NF-κB signaling is being widely investigated as an approach to treat such diseases.

### 1.2. NF-κB Signaling in Inflammatory Diseases

In mammals, the NF-κB family consists of the five structurally related transcription factors p50 (NF-κB1), p52 (NF-κB2), p65 (RelA), c-Rel and RelB [14,15]. There are three distinct pathways through which NF-κB signaling can occur: the canonical (or classical) pathway, the noncanonical (also nonclassical or alternative) pathway and the atypical signaling pathway [16]. These are classified by their different activating mechanisms (see Table 1).

**Figure 1:**
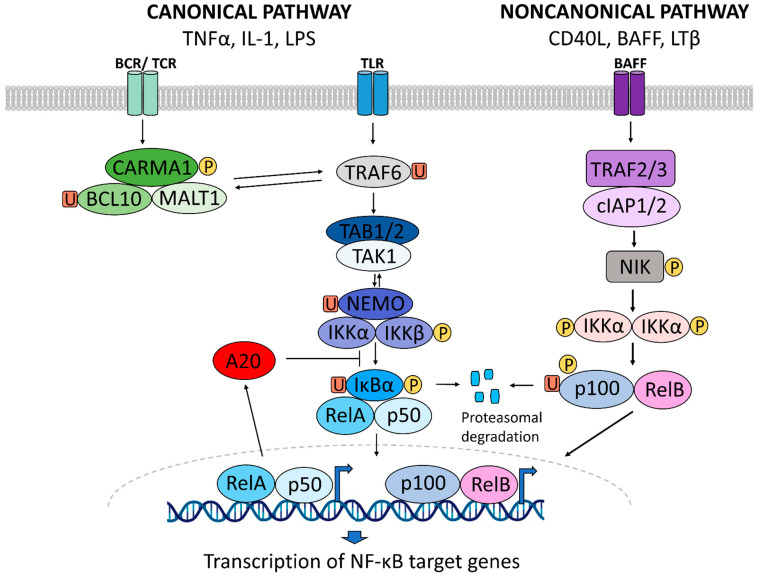
Activation of the canonical and noncanonical NF-κB signaling pathway.

The canonical NF-κB pathway can be activated by diverse stimuli such as TNF-α, IL-1 or LPS (Figure 1) [16]. Upon recognition of these ligands by their receptor, the IKK2 complex, consisting of IKKβ and NEMO (NF-κB essential modulator), is phosphorylated [13,20]. Subsequently, IκBα is phosphorylated [21,22,23], causing the ligation of ubiquitin chains to IκB, thereby tagging the inhibitor for proteasomal degradation [24]. Upon degradation of IκB, the nuclear localization sequences become unmasked, and the p65:p50 heterodimer can translocate from the cytoplasm into the nucleus where the transcription factor binds to the promoter of the primary response inflammatory genes including TNF or IL1β and initiates their transcription [5,16,25,26].

In contrast to the canonical pathway, the noncanonical pathway is IκB-independent [8] and is activated by a subset of members of the TNF cytokine family [13,27,28] (Figure 1). Under normal conditions, NF-κB-inducing kinase (NIK) is constantly ubiquitinated and degraded. Upon ligand binding, NIK is stabilized and consequently phosphorylates and activates IKKα. IKKα phosphorylates NF-κB subunit p100, which is subsequently ubiquitinated and cleaved to form p52 [27,29,30,31]. p52 proceeds to form a heterodimer with RelB, which translocates to the nucleus and binds DNA to induce transcription of target genes.

The canonical pathway is initiated by ligand binding to cytokine receptors such as the TNF-receptor or the IL-1 receptor and results in the activation of the IKK complex, consisting of IKKα, IKKβ and NEMO. This causes the phosphorylation (P) and the ubiquitination (U) of IκBα, targeting it for degradation by the 26S proteasome. The NF-κB dimer translocates to the nucleus where it activates the transcription of NF-κB target genes. This dimer can consist of p50/RelA, RelA/RelA, RelA/c-Rel, Rel/p52, c-Rel/c-Rel, p52/c-Rel, p50/c-Rel, p50/p50, RelB/p50 and RelB/p52, with the p50/RelA complex being the most common [32]. Binding of ligands to a subset of TNF receptor family members such as the CD40, BAFF or the LTβ receptor activates the noncanonical NF-κB pathway. Following ubiquitination of TRAF2/3 by cIAP1/2 at the receptor and subsequent degradation, NIK is stabilized. Activated NIK accumulates and phosphorylates IKKα, which in turn phosphorylates p100, causing it to be proteolytically processed to p52. RelB and p52 form a heterodimer which translocates to the nucleus to induce the transcription of target genes. Figure 1 was generated with BioRender and Powerpoint and summarizes experimental findings reviewed in [5,13,16,33,34,35].

Finally, atypical NF-κB signaling pathways are those that cannot be classified into either canonical or noncanonical signaling. Although each pathway is unique to the stimulus, atypical signaling is largely induced by genotoxic stress, such as UV damage or exposure to ROS. This signaling pathway can also be activated by casein kinase 2 or tyrosine kinases such as EGFR [18,36,37,38,39].

### 1.3. NF-κB Activity as a Druggable Target in Inflammatory Diseases

**Figure 2:**
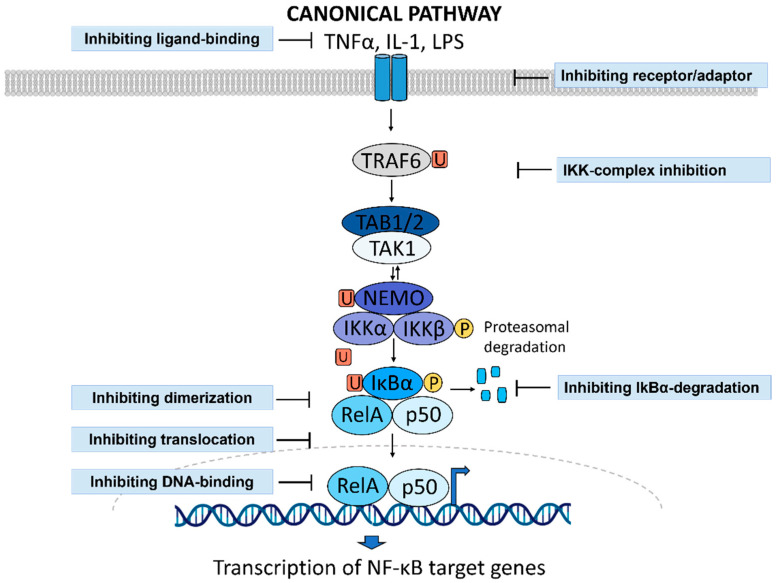
Strategies to inhibit NF-κB signaling.

NF-κB signaling can be modulated at different stages between receptor activation and the initiation of gene transcription [40]. Strategies for NF-κB inhibition include targeting the receptors, receptor adaptor proteins (e.g., BTK, IRAK, PI3K/AKT or c-IAP), the IKK complex or the ubiquitin-protease system to prevent the degradation of IκBα. Further, interfering with nuclear translocation, DNA binding or the initiation of transcription of NF-κB target genes are all attractive strategies to inhibit NF-κB signaling (Figure 2) [40,41].

NF-κB signaling can be inhibited by preventing the activation of receptors triggering NF-κB activation by using monoclonal antibodies or receptor antagonists. Targeting IKKα or IKKβ inhibits IκBα phosphorylation and ubiquitination. IκBα can be targeted directly, which can increase its expression. By inhibiting the proteasome, inhibitors can prevent the degradation of IκBα and the subsequent translocation of the p65/p50 dimer into the nucleus. Finally, inhibitors can interfere with nuclear translocation directly, as well as with DNA-binding or NF-κB target gene transcription. Figure 2 was generated with BioRender and Powerpoint.

Given the importance of NF-κB activity for the pathology of many human diseases, drug development efforts have identified a number of NF-κB inhibitors [42,43] that can be broadly categorized into recombinant proteins, peptides, natural products and synthetic compounds [41]. Despite hundreds of NF-κB inhibitors having been reported to date, few have found clinical application [40]. Therefore, this review aims to investigate drug repurposing as an alternative strategy to identify novel NF-κB inhibitors with anti-inflammatory properties.

## 2. Drug Repurposing to Identify NF-κB Inhibitors

### 2.1. Why Do We Need New Anti-Inflammatory Drugs?

Many common anti-inflammatory drugs have potentially serious side effects [44,45]. Disease-modifying antirheumatic drugs (DMARDs) require close monitoring due to the increased risk of infection and hepatotoxicity. Glucocorticoid treatment often results in glucocorticoid resistance and therefore is limited for long-term treatment [46,47]. TNF-blockers, a widely used intervention for autoimmune diseases such as MS or RA, can exacerbate MS symptoms as well as the frequency and the severity of MS attacks [48,49]. Furthermore, RA patients treated with TNF inhibitors can develop demyelinating lesions in the CNS or MS [50]. Finally, while a few compounds have been reported to have both anti-inflammatory and prorepair effects, very few have been investigated in clinical trials and none have received FDA-approval (Table 2). Instead, currently available anti-inflammatory drugs have been selected to reduce inflammatory mediator production and not necessarily selected for their ability to enhance tissue repair processes.

**Figure 3:**
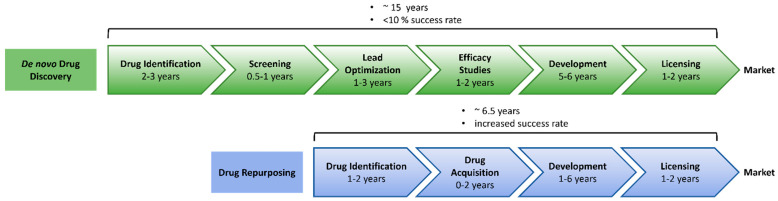
Timeline of conventional drug discovery versus drug repurposing. Figure adapted from [76] and generated with Powerpoint.

Drug repurposing (also referred to as drug repositioning, drug reprofiling or drug re-tasking) seeks to identify new uses for existing drugs or compounds outside the scope of their original indication [73]. Increasing costs of de novo drug discovery combined with long development timelines are major challenges in drug development. Bringing a new drug to the market has been estimated to take 15 years and cost an average of USD 2 billion to USD 3 billion. In contrast, drug repurposing is estimated to cost 10% that of de novo drug development. With an average timeline of 6.5 years, the repurposing process is much more time efficient [74,75] (Figure 3).

Drug repurposing is a time- and cost-effective alternative to de novo drug discovery. Available data collected during the various phases of development for the initial indication allows bypassing several steps of the conventional drug discovery process, thereby significantly reducing the risk of failure as well as time and costs involved in the procedure. Moreover, 90% of drug candidates fail in clinical trials due to safety and efficacy concerns. Because of extensive safety testing in preclinical animal models and in clinical trials [77,78], drug repurposing minimizes this risk of failure. Furthermore, drug repurposing offers the opportunity to rescue compounds that have undergone clinical testing and have good pharmacokinetic and safety profiles but have previously failed to achieve clinical approval due to a lack of efficacy in their original indications.

### 2.2. Approaches to Drug Repurposing

**Figure 4:**
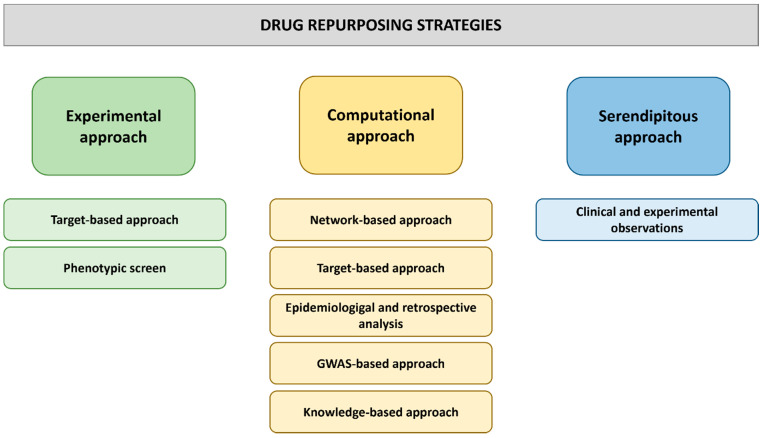
Drug repurposing approaches. Different experimental, computational and serendipitous strategies can be employed to identify promising drug repurposing candidates among existing drugs. This figure was generated with Powerpoint.

Many approaches to drug repurposing exist, including biological, experimental or computational approaches as well as combined approaches (Figure 4).

Initially, drug repurposing occurred when medicines were observed to have consistent unexpected off-target side effects in patients. This was the case with sildenafil, a drug developed to treat angina [57], which has since been successfully marketed by Pfizer to combat erectile dysfunction. After growing evidence highlighted the benefits of sildenafil treatment of pulmonary hypertension, the drug received approval to be further repurposed for the treatment of PAH [79]. Rare, serendipitous observations continue to be exploited, but this strategy has become a less reliable approach to drug repurposing, leading systematic approaches to dominate in recent years. In the next section of this review, different approaches to drug repurposing will be discussed, and specific examples of how they have been utilized to target NF-κB will be outlined.

## 3. Computer-Based Drug Repurposing Strategies

Computational drug repurposing strategies are screening approaches that are capable of testing thousands of candidate compounds at a rapid rate (Figure 4). Typically, these screens investigate libraries of drugs that have chemical structures or molecular targets similar to those of drugs already known to be active in the desired context. Molecular docking can predict previously unreported interactions of existing drugs with therapeutically relevant targets. Alternatively, screens can be performed to identify diseases with shared molecular targets and thus shared treatment options. Many successful drug repurposing efforts combine drug- and disease-based approaches [77,80]. Various databases, libraries and methods have been developed for computer-based drug repurposing (Table 3). Computational screening methods and resources for drug discovery and web-based resources for drug repurposing have been extensively reviewed recently [81,82].

A recent study identified thioridazinehydrochloride (TDZ) as a novel IKKβ inhibitor from a panel of FDA approved drugs [83]. TDZ is a first-generation antipsychotic used to treat symptoms of schizophrenia, other psychotic disorders, depressive disorders, behavioral disorders in children and geriatric psychoneurotic disorders. Mechanistically, TDZ is known to block dopamine-2 receptors in the mesolimbic pathway [84]. The drug repurposing strategy took a drug-based computational approach. Since de novo drug development has not resulted in the approval of any IKKβ inhibitors [33,85,86,87], the study aimed to repurpose existing drugs as IKKβ inhibitors by developing a computer-assisted structure-based drug repurposing strategy. A virtual screen using a subset of the ZINC database of FDA-approved drugs and a crystal structure of inhibitor-bound IKKβ revealed thioridazinehydrochloride (TDZ) as a potential IKKβ inhibitor. To validate the repurposing approach, TDZ was tested both in vitro and in vivo. TDZ was shown to inhibit IKKβ phosphorylation and IκBα degradation, thereby inhibiting NF-κB activity and resulting in the attenuation of inflammation in a mouse model of endotoxemia [83]. These findings validate the computer-aided drug repurposing approach to identify novel NF-κB inhibitors with anti-inflammatory properties, which can be further investigated for clinical benefit in NF-κB-dependent inflammatory diseases [83].

**Table 3:** List of databases, libraries and methods used in computer-based drug repurposing.

Resource	Description	References
Databases and Libraries		
Drug Repurposing Hub	Annotated library of FDA-approved drugs, drugs undergoing clinical trials, and preclinical tool compounds	[88]
DrugCentral	Online drug information resource containing 4714 drugs and 129,975 pharmaceuticals, providing up-to-date drug information, including a drug repositioning prioritization scheme for FDA-approved drugs	[89]
CheMBL	Database of bioactive molecules with druglike properties, containing chemical, bioactivity and genomic data to aid translation of genomic information into effective new drugs	[90,91]
ReDo_Trials Database	Database of active clincal trials investigating repurposed drugs for cancer therapy	[92]
RepoDB	Database of approved and failed drugs and their indications	[93]
ReFrame Database	Commercially available screening library of 12,000 molecules for use in high throughput cell-based repurposing assays	[94]
Zinc	Library of >700,000 small molecules for use in computational screening	[95]
COVID-19 Drug Repurposing Database (Excelra)	Commercially available database of approved drugs which can rapidly be entered into phase II or III trials against COVID-19	[96]
DrugRepurposing Online (Nimedicus)	Commercially available database of 9040 candidate repurposing compounds annotated with indications and mechanisms	[97]
PROMISCUOUS	Publicly available database of 25,000 drugs annotated with drug-protein, protein-protein interactions, drug structural similarity and known side-effects	[98]
**Methods**		
DeepDTNet	Deep learning system for identification of novel targets for drug repurposing in disease specific contexts	[99]
AOPDEF	Deep learning system identifying molecular targets among known drugs on two external validation sets	[100]
MBiRW	Computational method to identify novel indications for given drugs	[101]
KinderMiner	Text mining method to identify repurposing candidates	[102]
DrugQuest	Text mining method to identify simmilarities between DrugBank entries	[103]
Semantic Link Association Prediction (SLAP)	Statistical algorithm to predict novel drug-target pairs	[104]

### 3.1. Pharmacophore Modeling-Based Drug Repurposing

Pharmacophore modeling protocols have been established to repurpose drugs for different indications. Developing a pharmacophore model for target proteins linked to certain pathologies, taking into account molecular features required for the interaction of a ligand to the chosen protein target, can be used to generate pharmacophores with features predicted to cause strong interactions with the target or predict the binding and activity of molecules in a virtual screen [105]. The model is based on structural data available for the target or based on previously identified ligands [106]. This approach has been successfully employed to screen drug libraries, scoring the compounds against the pharmacophore model to identify drug repurposing candidates against inflammation [107,108], COVID-19 [109,110,111] or insulin resistance [112]. Early in the SARS-CoV-2 pandemic, the anti-inflammatory effect of thalidomide was suggested for the treatment of COVID-19 patients [113,114] To study the mechanism through which thalidomide ameliorates COVID-19 and to identify derivatives that could be promising candidates for treatment, a pharmacophore modeling-based repurposing approach was applied. This approach revealed the key protein targets involved in the regulation of the immune response by thalidomide. Processes that were affected by thalidomide were IkB phosphorylation, and MAPK signaling, among others. A transcriptome-based strategy was combined with gene expression analysis of cells treated with thalidomide or its derivative lenalidomide. This confirmed the previous results, as NF-κB and MAPK signaling were shown to be down-regulated [111].

### 3.2. Artificial Intelligence-Aided Drug Repurposing

Recently, drug repurposing strategies that use artificial intelligence (AI) to identify novel indications for existing drugs have been developed. For example, known drug-target interactions can be used to predict new interactions via an AI method called deepDTnet, which contains a heterogenous network of drugs, genes and diseases, including chemical, phenotypic and genomic data (Table 3) [99]. The method was tested using a library of FDA-approved small molecules and was shown to identify novel targets of a known drug using deep learning algorithms. This approach was successfully used to identify drugs that interact with ROR-γt, which is linked to autoimmune diseases such as MS [99]. The authors identified the FDA-approved topoisomerase inhibitor topotecan as a promising repurposing candidate, which was validated in the EAE mouse model in vivo [99]. The network-based arbitrary-order proximity embedded deep forest approach (AOPEDF) is based on deepDTnet and can accelerate target-based drug repurposing. Similar to deepDTnet, it integrates drug, disease and target data to identify new targets but seems more effective in predicting novel drug-target interactions [100].

## 4. Experimental Approaches to Drug Repurposing

Experiment-based drug repurposing approaches can be divided into target-based strategies and phenotypic screens (Figure 4). Target-based drug repurposing requires knowledge of the molecular target of candidate drugs, whereas phenotypic screens do not rely on extensive scientific knowledge of the mode of action of a drug or the molecular pathology of a disease [82,115,116,117].

### 4.1. Target-Based Approaches to Drug Repurposing

Given the role that tyrosine kinases such as CSFR-1, KIT, Lck and DDR play in RA pathology, multiple studies have investigated the effect of tyrosine kinase inhibitors (TKIs) in models of arthritis [118,119,120,121]. Dasatinib, a second-generation TKI used to treat chronic myeloid leukemia or Philadelphia chromosome-positive acute lymphoblastic leukemia [122,123], has been identified as a promising new therapeutic option for the treatment of RA. In one study, Guo et al. [71] investigated the effect of dasatinib on RA pathology due to its similar target profile to other TKIs imatinib and nilotinib, which were previously found to be effective in collagen induced arthritis (CIA) animal models [124,125]. Dasatinib reduced disease severity by attenuating the production of proinflammatory cytokines IL-1β, TNF-α and IL-6 in mice with CIA, while increasing anti-inflammatory IL-10 [71]. Moreover, dasatinib inhibited the migration and proliferation of human fibroblastlike synoviocytes (FLS), which in their activated state promote bone erosion based on their ability to secrete receptor activator of nuclear factor κB ligand (RANKL), thereby inducing osteoclast differentiation and bone destruction [71]. These findings validate dasatinib as an anti-inflammatory drug in a preclinical model that has the potential to be repurposed as an RA treatment.

### 4.2. Phenotypic Screening Approaches to Drug Repurposing in Cell Lines and Model Organisms

Preclinical drug identification and development traditionally relies on cell-based assays to identify and optimize promising lead compounds. With nine out of ten drugs entering clinical trials failing to achieve FDA approval [126,127], there is a need for reliable assays to test the safety and effectiveness of drugs in early drug development stages.

In order to identify FDA-approved drugs that promote remyelination in MS, Mei et al. developed a high-throughput functional screening assay using micropillar arrays, which allow for the detection and quantification of myelin wrapping [57]. The screen identified clemastine fumarate, an H_1_-antihistamine that is used to treat allergic reactions. Clemastine promotes oligodendrocyte precursor cell differentiation in animal models and human cells [52,57,58]. As only differentiated oligodendrocytes can produce myelin [128], this differentiation process induced by clemastine was linked to an increase in remyelination in a variety of animal models [52,55,56,57,58,61], which was confirmed to be specifically due to increased oligodendrocyte differentiation [58]. Furthermore, it inhibited the production of proinflammatory cytokines, microglial M1-like activation and astrocyte loss in mice with depressionlike symptoms and a mouse model of ALS [51,59]. Studies have linked the anti-inflammatory activity of clemastine to its ability to inhibit NF-κB [54,60]. A phase II clinical trial recently demonstrated the ability of clemastine to promote myelin repair in patients with relapsing MS [53].

Although many cell-based assays allow for high throughput screening, results obtained from in vitro testing on human cells or tissues have limited reliability in terms of the effect of the drug on a whole organism. Therefore, automated, high-throughput, quantitative in vivo screens have been developed, with *Danio* zebrafish becoming an increasingly popular model organism [129] due to their increased throughput screening capacity in comparison to mice and the resemblance of their immune system to that of humans [120,121]. Several zebrafish inflammation models have been developed, which have been successfully used to identify and study drugs with anti-inflammatory properties [129]. Hall et al. demonstrated the potential of using in vivo zebrafish neutrophil migration assay in screening for novel anti-inflammatories [90]. The assay, which assesses the recruitment of neutrophils to tail fin injury as a model of acute inflammation, was applied to identify previously unknown anti-inflammatory properties of approved drugs in a high-throughput screen. The anti-inflammatory activity of the 10 most potent repurposing candidates was subsequently tested in a mouse model of atopic dermatitis, in which they potently inhibited dermatitis-related inflammation [130].

Zebrafish embryos are also a useful model organism in drug development screens. Their innate immune system develops early in embryogenesis, and as early as 26 h after fertilization, phagocytosis and ROS production can be detected in embryonic macrophages [131,132]. Not until later stages of development does the adaptive immune system mature, therefore making it possible to study both arms of the immune response [133,134].

Furthermore, in zebrafish (*Danio rerio*), the blood brain barrier (BBB) is not developed until 3–10 days postfertilization, with tight junctions forming after day 5. Therefore, drugs added into water can cross the BBB, allowing modulatory effects on zebrafish behavior to be studied [135]. In the first study investigating the behavioral profile of zebrafish, Rihel et al. were able to classify drugs in a high-throughput functional screen by analyzing the rest/wake cycle of fish [136]. For example, anti-inflammatory drugs including glucocorticoids and NSAIDs coclustered by promoting a unique sleep/wake behavioral fingerprint. There is exciting potential to use behavioral fingerprints to identify anti-inflammatory activity of existing drugs.

## 5. NF-κB as a Potential Target for Drug Development in CNS Inflammation

**Figure 5:**
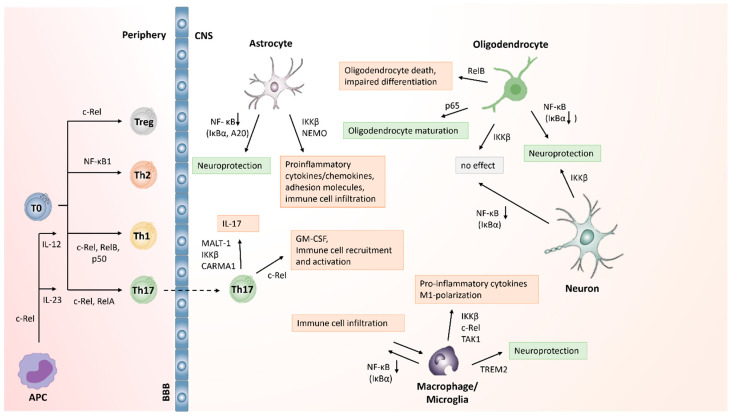
Cell-type specific role of NF-κB signaling in MS and EAE. Elements from this figure were adapted from [87,182]. This figure was generated with BioRender.

Multiple sclerosis is an autoimmune disorder that causes chronic inflammation in the central nervous system (CNS). It is the most widespread disabling neurological disease in young adults and results in physical or cognitive disorders [137]. MS pathology is the result of immune cells infiltrating the CNS, releasing cytokines and other inflammatory mediators leading to the destruction of myelin sheath, the reduction of oligodendrocyte numbers and finally axon degeneration [138]. Canonical and noncanonical NF-κB signaling play an important role in MS pathology in both innate and adaptive immune cells (Figure 5 and Table 4). Genomewide association studies (GWAS) have correlated central components of the NF-κB pathway with an increased risk of developing MS [139,140,141].

### 5.1. NF-κB Activation in T Cells in MS/EAE

NF-κB drives the expression of proinflammatory mediators, which induces the differentiation of naïve CD4+ T cells towards proinflammatory Th1 and Th17 cells. On the other hand, NF-κB is also required for the Treg differentiation [140,142,143]. In addition, NF-κB activity increases the expression of adhesion molecules, enabling infiltrating inflammatory T cells to cross the blood brain barrier (BBB) [144,145,146,147]. Deficiency in NF-κB signaling components such as NIK, p50, IKK2, RelA or c-Rel decreases T cell differentiation or activation and protects from EAE (experimental autoimmune encephalitis) [148,149,150,151,152,153,154]. EAE is a commonly used animal model of MS and one of the oldest models of immunopathology. Animals present with neuroinflammation, destruction of myelin sheath, axon damage and gliosis, which are key features observed in MS pathology, but also undergo resolution of inflammation and remyelination. Therefore, EAE is a complex model system for testing and development of potential MS medications [155]. Unlike in MS, the disease is not initiated through the production of myelin-directed autoantibodies but requires induction. Active immunization is induced with myelin antigens or spinal cord homogenate in combination with an adjuvant, whereas passive immunization is induced through the adoptive transfer of myelin-specific CD4+ T cells from a donor animal [156,157].

### 5.2. Role of NF-κB Activation in Macrophages and Microglia in MS

NF-κB activation in macrophages or microglia in MS and EAE exacerbates inflammation by promoting the production of proinflammatory mediators. Therefore, additional macrophages/microglia are activated, enhancing inflammation even further [158,159]. In mice with a myeloid-specific conditional IκBα knockout, constitutive NF-κB activity exacerbates the severity of EAE. This is due to an increase in T cell and macrophage/microglia infiltration. In addition, inducible nitric oxide synthase (iNOS) expression promoted by NF-κB activity is increased as well as the production of myeloid-derived proinflammatory cytokines [160].

Research using myeloid cells from mice with conditional knockouts of IKKβ demonstrates the role of the kinase in demyelination and neurodegeneration. IKKβ depletion specifically in microglia and macrophages decreases T cell and macrophage infiltration, the permeability of the BBB, the transcription of proinflammatory genes as well as neuroinflammation, demyelination and EAE severity. In addition, the amount of Th1 and Th17 cells is reduced, whereas the percentage of Treg cells increases in the spinal cord [161,162]. To determine the role of microglia independently from peripheral macrophages, Goldmann et al. developed a microglia-specific conditional TAK1 knockout mouse model. TAK1 depletion inhibited the NF-κB pathway and reduced damage to the axons and myelin sheaths as well as overall CNS inflammation [163]. Taken together, these results demonstrate the proinflammatory role of NF-κB activity in macrophages and microglia in EAE.

However, NF-κB expression in macrophages might also have a neuroprotective effect. When TREM2, a target of NF-κB, was overexpressed in bone-marrow derived myeloid precursor cells which were applied to EAE mice, an increase in the production of anti-inflammatory cytokines was observed. This was accompanied by the amelioration of EAE symptoms and the reduction of demyelination and axon damage, while increased phagocytosis led to the clearance of destroyed myelin [164].

**Table 4:** Role of NF-κB activity in different cell types in MS/EAE.

Cell Type	Genotypic Alteration in NF-κB Signaling	Effect on Neuroinflammation	References
T cells	IKKβ deficient T cells	Resistance to EAE, impaired autoreactive T cell activation and expansion	[149]
	p50 deficient	Attenuated EAE incidence and severity, impaired Th1 and Th2 differentiation	[150]
	c-Rel deficient	Resistance to EAE, defective Th1 and Th17 development	[148,151]
	MALT1 deficient	Protection from EAE, absence of demyelination, proinflammatory cytokines and immune cell infiltration into spinal cord. Effector function of autoreactive Th17 cells impaired	[165,166]
	CARMA1 deficient	Resistance to EAE, impaired Th17 differentiation	[167]
	IκBαΔN	Resistance to EAE, reduced Th17 differentiation	[167]
	NIK deficient	Protection from EAE due to DC function and independent from CD4+ T cell function	[168]
	NIK deficient	Resistance to EAE, impaired Th17 differentiation	[152]
	NIK deficient T cells	Attenuation of EAE, reduced generation of Th1 and Th17 cells, reduced immune cell infiltration	[153]
Macrophages/ Microglia	IκBα deficient	Exacerbated EAE, increased immune cell infiltration and myeloid-derived proinflammatory cytokines	[160]
	IKKβ deficient macrophages/ microglia	Attenuation of EAE, reduced immune cell infiltration, production of proinflammatory cytokines and permeability of the BBB. Increase in Tregs and decrease of Th1 and Th17 cells	[161,162]
	TAK1 deficient microglia	Reduced CNS inflammation and neurodegeneration, NF-κB inhibition	[163]
	TREM2 overexpressing myeloid precursor cells	Attenuation of EAE, reduced neurodegeneration, increase in anti-inflammatory cytokines and phagocytosis	[164]
	A20 deficient microglia	Aggravated EAE, Nrp3 inflammasome activation, increase in immune cell infiltration and proinflammatory cytokine production	[169]
Astrocytes	IκBα overexpressing astrocytes	Attenuation of EAE, decreased immune cell infiltration and production of proinflammatory cytokines	[170,171,172]
	IKKβ deficient astrocytes	Protection from myelin loss in cuprizone-induced inflammation model	[173]
	A20 deficient astrocytes	Aggravated EAE, increase in immune cell infiltration and proinflammatory cytokine production	[174,175]
Oligodendrocytes	IκBαΔN in oligodendrocytes	Aggravated EAE, reduced remyelination and oligodendrocyte death in cuprizone-induced inflammation model	[176]
	IKKβ deficient oligodendrocytes	No protection from demyelination in cuprizone-induced inflammation model	[173]
Neurons	IKKβ deficient neurons	Aggravated EAE, increased Th1 infiltration and proinflammatory cytokine production. Reduced production of neuroprotective factors	[177]
	IκBα overexpressing neurons	No effect on EAE progression or inflammation	[178]

### 5.3. NF-κB Activation in CNS Cells in MS

In the CNS, the conditional deletion of NEMO or IKKβ in nonmicroglial cells (astrocytes, oligodendrocytes and neurons) has an anti-inflammatory effect. This is abrogated when IKK1 is eliminated in the cells, suggesting that NF-κB activation through the canonical NF-κB pathway plays a role in CNS cells in EAE. However, the model made it impossible to determine the contribution of the individual nonmicroglial CNS cell types to the proinflammatory function [179].

In astrocytes, NF-κB signaling seems to be an important driver of their proinflammatory activity in EAE. An improvement of EAE symptoms, a decrease in proinflammatory gene expression and immune cell infiltration was detected in mice with astrocyte-specific overexpression of IκBα, resulting in the inhibition of NF-κB signaling. In addition, the transgenic mice also showed improved recovery from EAE [170,171,172]. The ubiquitin-modifying protein A20, which inhibits NF-κB activity, could be an important regulator of NF-κB signaling in EAE. The deletion of A20 in astrocytes leads to increased production of proinflammatory cytokines and immune cell recruitment, resulting in aggravated EAE disease severity [174,175]. In line with this, the astrocyte-specific deletion of IKK2 protects mice from myelin loss in a cuprizone-induced inflammation model [173].

In contrast to astrocytes, NF-κB activation has a protective effect on oligodendrocytes in multiple models of MS. The overexpression of the NF-κB repressor IκBα in oligodendrocytes increases oligodendrocyte death and hypomyelination in IFN-γ expressing mice and causes the failure of remyelination and the death of IFN-γ induced remyelinating oligodendrocytes in the cuprizone model. NF-κB inhibition also increases the susceptibility of the IκBαΔN mice to EAE [176]. However, oligodendrocyte-specific IKK2 depletion does not protect mice from demyelination in the cuprizone model, which indicates that IKK2-mediated NF-κB activation does not play an important role in remyelination [173]. These results contrast with earlier findings that NF-κB activity was detected in oligodendrocytes and microglia/macrophages on the edge of inactive MS lesions but not silent MS plaques, which suggests that oligodendrocytes are involved in tissue repair [180]. In addition, mice with a conditional knockout of RelB in oligodendrocytes display enhanced p65 NF-κB activity and survival of mature oligodendrocytes, resulting in reduced EAE severity. Increased p65 activation was previously shown to promote oligodendrocyte survival in inflammation and was suggested to be due to the lack of RelB-mediated inhibition [181].

NF-κB has a neuroprotective effect on neurons in EAE. Mice with a deletion of IKKβ specific to Ca^2+^/calmodulin-dependent kinase IIα-expressing neurons developed severe EAE, characterized by axon loss, Th1 cell infiltration, reduced production of neuroprotective factors in the CNS and NK cell recruitment as well as the up-regulation of proinflammatory cytokine and chemokine expression [177]. However, Lee and colleagues were unable to detect any effect of NF-κB activity on neurodegeneration in EAE. The conditional overexpression of IκBα, a repressor of NF-κB, in neurons did not influence EAE progression, inflammation or axon degeneration. The authors speculated that the deletion of IKKβ upstream of IκBα may have more widespread effects or that unknown targets of IKK2, besides IκBα, may be involved in neuroprotection in EAE [178].

These results demonstrate that NF-κB activity in inflammatory T cells, macrophages and microglia as well as astrocytes has proinflammatory effects and aggravates MS and EAE pathology. However, some data also indicate that NF-κB signaling might have an anti-inflammatory effect in oligodendrocytes and neurons and possibly in macrophages in some cases, which can protect against neurodegeneration (Figure 5). The role of NF-κB in MS and EAE seems to be highly dependent on the specific cell type (Table 4).

NF-κB activity plays a key role in the development and progression of inflammation in MS and EAE. It causes Th17 cells, macrophages and microglia and astrocytes to produce an increased amount of proinflammatory cytokines, chemokines and adhesion molecules. Furthermore, the transcription factor leads to the recruitment of more immune cells, thereby exacerbating neuroinflammation. Conversely, NF-κB activity can also have a neuroprotective effect in MS and EAE, depending on the cell type. Proinflammatory cell-specific roles of NF-κB in MS/EAE are shown in red boxes in Figure 5, whereas anti-inflammatory and protective effects are shown in green. Therefore, the role of NF-κB in MS strongly depends on the cell type, which needs to be considered when developing treatment strategies.

### 5.4. Repurposing NF-κB Inhibitors to Treat CNS Inflammation

The anti-inflammatory effect of many FDA-approved drugs used to treat MS is thought to be linked in part to their ability to inhibit NF-κB signaling [87,159]. These drugs were not developed as specific NF-κB inhibitors; however, they were found to interfere with NF-κB activation at different stages of the pathway. Therefore, finding approved drugs with the ability to inhibit NF-κB activity for drug repurposing in MS seems like a promising strategy to identify new treatment options (Table 5).

Imatinib mesylate is a tyrosine kinase inhibitor targeting Bcr-Abl, first approved for the treatment of chronic myeloid leukemia (CML) in 2003. Since then, imatinib has shown to also be a potent inhibitor of NF-κB signaling and inflammation in vivo and in vitro [183,184]. This effect was linked to a reduction of IκB phosphorylation as well as DNA binding of NF-κB in human myeloid cells [185]. Imatinib is currently being tested in a phase II clinical trial to compare the effects of the drug to those of standard treatment in patients with relapsing multiple sclerosis (Trial number NCT03674099).

While repurposing NF-κB inhibitors to treat MS seems like a promising strategy, certain limitations of this approach must be considered. A variety of studies highlight the important role of NF-κB activity in the development and progression of MS and EAE. In immune cells such as macrophages, microglia and T cells, NF-κB signaling promotes the production of proinflammatory cytokines, which enhances inflammation and contributes to tissue damage and disease progression. However, the protective role of NF-κB activation in oligodendrocytes and neurons has also been demonstrated [154]. NF-κB plays different roles in different cell types in MS. Therefore, broad inhibition of NF-κB activity does not seem like an ideal therapy strategy for MS. Tight regulation of NF-κB signaling in a cell-type specific manner will be necessary to avoid toxic side effects or the impairment of general immune function.

## 6. NF-κB as a Potential Target for Drug Development in Joint Inflammation

Rheumatoid arthritis (RA) is a chronic systemic autoimmune disorder, in which the lining of the synovial joints is degraded due to immune cell infiltration and inflammation [13,215]. As the disease progresses, it results in the destruction of cartilage and bone, leading to disability. Systemic inflammation associated with RA can cause premature death, often due to cardiovascular disease, with 0.5 to 1% of the population being affected in 2002 [215,216]. There are currently no drugs available to cure RA. To decrease disease activity and treat joint stiffness and pain, nonsteroidal anti-inflammatory drugs and corticosteroids are prescribed. However, these treatments do not affect disease progression [215].

**Figure 6:**
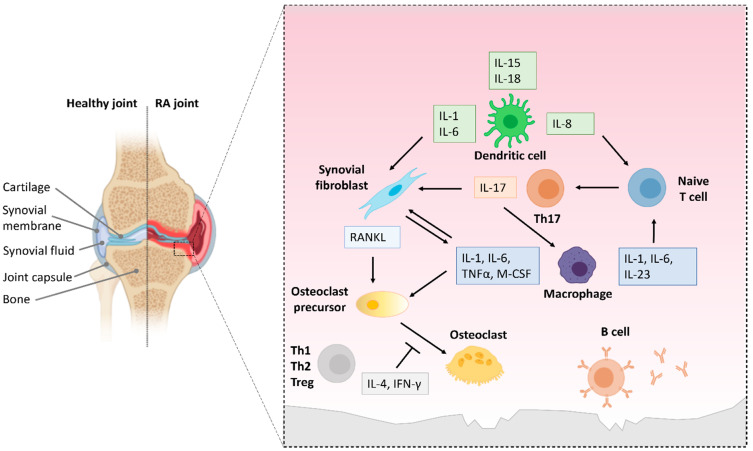
The role of NF-κB activity in the pathology of RA. This figure summarizes experimental findings reviewed in [227,230,231].

NF-κB has been identified as a key player in RA in both human and animal models (Figure 6) [217,218,219]. Multiple studies have found increased NF-κB activity in inflamed synovial tissue in human patients with RA [217,218,219]. More specifically, p50 and p65 were detected in CD14+ macrophages in synovial tissue from RA patients, which highlights the contribution of NF-κB activation in macrophages and macrophage-derived cytokines to RA pathology [220].

In animal models of RA, NF-κB is activated in the synovium [221]. NF-κB inhibition reduces proinflammatory gene transcription, resulting in the attenuation of inflammation [221,222,223]. Interestingly, NF-κB activation can be detected before the onset of clinical symptoms. As the disease progresses, NF-κB activity increases [224]. These findings demonstrate that inhibiting the NF-κB pathway could be a promising target to treat RA.

### 6.1. NF-κB Activation in Innate Immune Cells in RA

In innate immune cells, the NF-κB pathway promotes the expression of proinflammatory mediators such as the cytokines IL-1, IL-6 and TNF-α, as well as adhesion molecules required for leukocyte migration such as VCAM1 and ICAM1 [225]. Besides playing a key role in inflammatory processes, macrophage-monocyte precursors are directly responsible for bone destruction observed in RA (Figure 6). Triggered by M-CSF, RANK expression is induced on the precursor cells, causing the innate immune cells to differentiate into osteoclasts and as such contribute to bone loss, since the balance between bone degradation and bone formation is lost [226,227]. Furthermore, many of the cytokines produced by monocytes/macrophages activate NF-κB signaling in other innate immune cells as well as in fibroblasts, resulting in the expression of additional proinflammatory mediators, the recruitment of proinflammatory immune cells and the exacerbation of inflammation [228]. In RA, monocytes/macrophages amplify inflammation further through the production of cytokines like IL-1, IL-6 or IL-23, which induce the differentiation of proinflammatory Th17 cells that play a key role in driving RA pathogenesis [229].

Bone destruction in RA is mediated by the interplay of macrophages, fibroblasts, DCs, B cells and infiltrating T cells in the synovium [227,231,232]. NF-κB activation in those cells can trigger proinflammatory responses, exacerbating disease pathology. In the synovial membrane, NF-κB activation causes proinflammatory T cells, macrophages and synovial fibroblasts to produce proinflammatory mediators, creating positive feedback loops. This results in the progression of inflammation and bone erosion. The arrows in Figure 6 indicate the effect of cytokines/proteins up-regulated upon NF-κB activation on other cells, thus exacerbating bone destruction in RA.

### 6.2. NF-κB Activation in T and B Cells in RA

Besides indirectly promoting the development of proinflammatory T cells through macrophage/monocyte activation, NF-κB also directly regulates the transcription of lineage factors of T cells [233,234,235] (Figure 6). In the serum and the synovial fluid of RA patients, elevated levels of BAFF (B cell activating factor) were detected, which correlate with the severity of the disease. BAFF binds to BCMA, which then activates the canonical and the noncanonical NF-κB pathway [236,237]. In turn, noncanonical NF-κB signaling was suggested to contribute to this increase in BAFF levels. This promotes the survival of B cells that react to self-antigens and produce autoantibodies, thereby accelerating RA pathogenesis [238,239].

Furthermore, NF-κB activity plays a role in B and T cell activation, proliferation and the differentiation of DCs [240,241,242] (Figure 6). The activation of the canonical and the noncanonical NF-κB pathway in DCs has different outcomes with regards to inflammation. While canonical NF-κB signaling results in the production of inflammatory cytokines early on in the inflammatory process, the noncanonical NF-κB pathway induces Treg function by promoting the expression of indoleamine 2,3-dioxygenase (IDO), thus modulating inflammation by reducing the production of proinflammatory cytokines and promoting Treg development by DCs [243]. In addition, NIK and the p50-RelB dimer induce Treg survival, proliferation and activation [244,245]. However, the noncanonical NF-κB pathway, has also been linked to proinflammatory responses. NIK is required in DCs to induce Th1 and Th17 proliferation, differentiation and effector function, thereby promoting the development of autoimmune disease [168]. In B cells, activation of the noncanonical pathway by BAFF is required for proliferation and survival as well as antibody production and thus drives chronic inflammation in the synovium in RA [246].

### 6.3. Repurposing NF-κB Inhibitors to Treat Joint Inflammation

Drug repurposing efforts have identified medicines that have anti-inflammatory effects in RA animal models by inhibiting NF-κB signaling, making them promising candidates for further studies (see Table 5).

Bruton’s tyrosine kinase (BTK) has emerged in recent years as a therapeutic target for the treatment of inflammatory disease. Originally discovered for its critical role in B cell development, and notable as the cause of the primary immunodeficiency X-linked agammaglobulinemia (XLA), in which patients harbor a loss-of-function mutation, BTK is also highly expressed in monocytes, macrophages and neutrophils [247]. In these myeloid cells, BTK has been demonstrated to play a role in NF-κB and NLRP3-inflammasome activation [193,247,248,249]. The BTK inhibitor ibrutinib demonstrates anti-inflammatory activity in preclinical models of RA [190,192], sepsis [191] and diabetes [193].

An alternative approach to inhibit NF-κB signaling is to target the proteasome using proteasome inhibitors (PIs). Bortezomib is a proteasome inhibitor that is clinically used to treat multiple myeloma [250]. The drug forms covalent adducts with the threonine residues in the active site of the proteasome and has proven to be an effective anti-inflammatory treatment in autoantibody-mediated immune disease models including MS, RA or colitis [194,195,196,197,199]. In addition to selectively destroying plasma cells in antibody-mediated autoimmune disorders, bortezomib also promotes the differentiation and activation of osteoblasts in multiple myeloma patients [250,251,252,253]. In patients with multiple myeloma and RA, bortezomib improved the condition of the joints [197,198]. In addition, bortezomib prevents the release of cytokines induced by NF-κB, and promotes apoptosis in T effector cells in RA patients [201].

## 7. Drug Repurposing for Targeting Inflammation in COVID-19 Pneumonia

The coronavirus disease 2019 (COVID-19) pandemic, caused by SARS-CoV-2, created an urgent need for both novel antiviral and anti-inflammatory drugs. In severe cases, SARS-CoV-2 induced pneumonia can result in life-threatening acute respiratory distress syndrome (ARDS) [254,255]. The most prominent cause of death in COVID-19 patients is a hyperinflammatory immune response characterized by production of proinflammatory cytokines, which causes tissue damage, mostly in the lung [254,256,257]. Severe COVID-19 is linked to hyperactivation of NF-κB signaling, which causes the increased release of proinflammatory molecules such as IL-1, IL-6, IL-12, IL-17, IFN-γ or TNF-α by infiltrating immune cells [254,256,258].

Several studies have shown that SARS-CoV infection triggers NF-κB activation. The viral nucleocapsid protein causes dose-dependent activation of NF-κB in SARS-CoV susceptible Vero E6 cells [259], and the nucleocapsid protein of SARS-CoV-2 was shown to recruit TAK1 and the IKK complex in HEK293T cells [260] to induce NF-κB signaling. The spike protein of SARS-CoV as found to induce activation and translocation of NF-κB in human PBMCs and THP-1 cells in vitro, which resulted in a dose-dependent increase in proinflammatory gene transcription. This effect was reversed by TPCK, a specific NF-κB inhibitor [261]. In vivo studies confirmed that the inhibition of NF-κB in SARS-CoV infected mice with severe acute respiratory syndrome reduced inflammation and lung pathology and significantly increased survival rates [262].

During coronavirus infection, the NF-κB pathway gets activated through viral pattern recognition receptors via MyD88, resulting in the induction of transcription of proinflammatory mediators [263]. Accordingly, in MyD88^−/−^ mice infected with SARS-CoV, a reduction of infiltrating monocytes and macrophages in early disease stages was observed, along with the persisting absence of cytokine and chemokine production [264]. Furthermore, another study demonstrated that the spike protein of the virus induces NF-κB activation in a TLR2 and MyD88-dependent manner, resulting in the production of proinflammatory cytokines and chemokines by human and murine macrophages [265]. These results strongly support the notion that identifying NF-κB inhibitors with anti-inflammatory properties could help mitigate hyperinflammation and attenuate disease severity in COVID-19 patients (Table 5).

Dexamethasone, a glucocorticoid commonly used to treat inflammatory diseases such as RA, is known to inhibit NF-κB signaling and the production of proinflammatory cytokines by promoting the overexpression of IκBα [207,208]. The RECOVERY trial found that dexamethasone reduced mortality in hospitalized COVID-19 patients in later but not earlier stages of the disease [205,206]. The authors concluded that at later stages of the disease, hyperinflammatory events dominate, which may explain why dexamethasone is more effective in these patients [205]. At earlier stages of COVID-19, viral replication needs to be limited by an appropriate antiviral immune response. Previous studies have shown that glucocorticoid treatment at early stages of the disease dampens the immune response and hence increases the risk of enhanced viral replication. This might explain why dexamethasone treatment might be a more attractive treatment option for patients with severe disease pathology [258,266]. The impressive therapeutic benefits of dexamethasone in severe COVID-19 have been demonstrated extensively and the scientific literature suggests a strong link between its anti-inflammatory effects and its ability to inhibit NF-κB signaling. However, its effect has not yet been exclusively linked to NF-κB inhibition.

Due to their anti-inflammatory properties, other RA drugs and kinase inhibitors were considered as potential candidates to be repurposed for the treatment of COVID-19 [267,268]. The IL-1 receptor antagonist anakinra reduces proinflammatory cytokine production by preventing NF-κB activation and has been shown to be effective in treating patients who exhibit hyperinflammation [214]. The effectiveness of anakinra to treat COVID-19 was tested in clinical trials. The drug reduced hyperinflammation and improved clinical signs of COVID-19 as well as mortality rates [209,210,211,212,213]. These examples of successful drug repurposing demonstrate that this strategy is a time- and cost-efficient way to discover drugs with useful anti-inflammatory properties in a short period of time.

## 8. Problems with Progressing Repurposed Drugs to Clinical Applications

The effectiveness and safety of drugs identified by repurposing still need to be carefully assessed before they can be approved for a new indication. A recent, well-publicized example of rushed approval of a drug repurposing candidate is the use of hydroxychloroquine (HCQ) in COVID-19 infected patients. HCQ is commonly used for the treatment of nonresistant malaria, RA and systemic lupus erythematosus. Its anti-inflammatory properties are attributed to the inhibition of NF-κB signaling and NLRP3 inflammasome inhibition, reducing the production of proinflammatory cytokines and macrophage and neutrophil infiltration in animal models of renal ischemia/reperfusion injury and immunoglobulin A nephropathy [269,270,271].

Early in the COVID pandemic, HCQ was suggested to have anti-inflammatory effects on SARS-CoV-2 infection in vitro [272,273]. A small open-label nonrandomized trial associated HCQ with lower viral load in patients hospitalized with COVID-19 [274], causing the FDA to issue an early use authorization (EUA) [275,276]. However, multiple studies subsequently showed that HCQ had no beneficial effect for COVID-19 patients or when used as pre or postexposure prophylaxis [277,278,279,280,281,282,283,284,285,286,287]. On the contrary, studies reported serious cardiac adverse events attributed to HCQ treatment [288,289], which is of great concern as COVID-19 is associated with cardiac complications [290,291]. Consequently, the FDA revoked the EUA [292]. The case of HCQ as treatment for COVID-19 demonstrates that extensive high-quality clinical trials, not just small, underpowered nonrandomized studies or unreliable observational data, are necessary to investigate the safety and efficacy of a drug before it can receive approval to be repurposed for the treatment of a different disease.

## 9. Conclusions and Future Prospects

The NF-κB pathway is a key player in many inflammatory diseases. Modulating NF-κB activity is a promising target for the treatment of inflammation, as many FDA approved drugs or drugs currently in clinical trials inhibit NF-κB signaling in addition to their originally identified mechanisms. In this review, we have shown how different drug repurposing strategies can be used to identify new modes of action for existing drugs as well as to indicate new applications for these drugs in inflammatory diseases linked to NF-κB signaling.

The SARS-CoV-2 pandemic has highlighted the advantages and limitations of drug repurposing, While drug repurposing offers higher success rates and is more time- and cost-efficient than de novo drug discovery, it is crucial to carefully assess candidate drugs in well-designed and sufficiently powered clinical trials before they receive approval for any new indication. Even though off-target effects of repurposed drug candidates may be well known, they still need to be carefully monitored, particularly when the molecular target for the new indication differs from that of the current indication.

Furthermore, many NF-κB inhibitors have been tested only in specific cell types using a limited number of stimuli such as LPS or TNFα. In addition, concentrations used in many assays that were necessary to achieve sufficient NF-κB inhibition are often higher than what would be feasible in vivo [42]. It will therefore be necessary to use more pathologically relevant stimuli and to carry out drug screening in whole organisms such as zebrafish where possible.

Many components of the NF-κB pathway overlap with other pathways, making the development of specific NF-κB inhibitors a complex task. This problem could be overcome by using combinations of inhibitors targeting different steps in the NF-κB pathway in low concentrations [42]. Furthermore, the transcription of specific target genes could be modulated to achieve the desired specificity. One example is the nuclear modification of RelA. Phosphorylation, ubiquitination and acetylation at certain sites can modulate the transactivation activity of the transcription factor and influence its DNA binding ability and/or protein stability [293]. The specificity of NF-κB signaling is further determined by the interaction of NF-κB dimers with the DNA and promoters/enhancers [294]. Therefore, interfering with nuclear modification of NF-κB could be a promising strategy to inhibit a specific set of NF-κB target genes while minimizing the effect on other signaling pathways. However, NF-κB has many essential physiological functions, which need to be preserved while its pathological effect is inhibited. Global NF-κB suppression is associated with severe toxicities in animal models and in humans [295,296,297,298].

In addition to the cell-type specific role of NF-κB in diseases such as MS, systemic NF-κB inhibition may result in multiple unwanted side effects, especially if employed as a long-term treatment. Therefore, identifying NF-κB inhibitors that predominantly target certain cell types over others might lead to a more favorable outcome in inflammatory diseases. Recently, the “sneaking ligand” (SL) approach was proposed for specific NF-κB inhibition: These ligands consist of an N-terminal domain, which binds to the cell surface, a translocation domain and a C’-terminal effector peptide, which interacts with its cytoplasmic ligand to modulate NF-κB signaling. This was validated in E-selectin-expressing endothelial cells, in which IKK complex assembly was inhibited in vitro and in vivo, resulting in the reduction of NF-κB activity specifically in E-selectin-expressing cells as well as the attenuation of experimental arthritis (STIA and AIA) in mice [230,299]. To achieve cell-specific NF-κB inhibition, several targeted drug delivery systems have been developed that combine ligands that are linked to cargo. Recombinant monoclonal antibodies can be coupled to a drug to achieve cell specificity. This approach is already being tested in clinical trials for application in various autoimmune diseases. Furthermore, aptamers that recognize specific patterns on a receptor can be conjugated to small molecules and peptide-drug conjugates, or peptide-modified nanocarriers can help target drugs to specific tissues or cells [35]. Many of these strategies are currently being explored in clinical trials and could be applied for specific NF-κB inhibition.

NF-κB mediates both pro and anti-inflammatory effects in MS depending on the cell-type. As most currently used or repurposed, NF-κB inhibitors inhibit their target more systemically. Their application may be more suitable to treating systemic inflammation (e.g., sepsis) or diseases in which NF-κB inhibition is more clearly linked only to proinflammatory processes (e.g., COVID-19, RA).

## Figures and Tables

**Table 1:** Stimuli and receptors triggering NF-κB activation [17,18,19].

Stimulus	Receptor	NF-κB Pathway
LPS	TLR4	Canonical
TNF-α	TNFR1	Canonical
IL-1	IL-1R	Canonical
BAFF	BAFFR	Noncanonical
CD40L	CD40	Noncanonical
RANKL	RANK	Noncanonical
LTβ	LTβR	Noncanonical
TNF	TNFR2	Canonical/Noncanonical
TWEAK	Fn14	Canonical/Noncanonical
EGF	EGFR	Atypical
UV	CK2	Atypical

**Table 2:** Compounds with reported anti-inflammatory and prorepair properties. Few compounds have been reported that both inhibit inflammation and promote repair processes. While some have been investigated in clinical trials, none have received FDA-approval: SCI = spinal cord injury; IR = ischemia/reperfusion; TBI = traumatic brain injury.

Compound	Original Indication	New Indication	Comments	References
Clemastine fumarate	Allergic reactions	MS	Promotes oligodendrocyte precursor cell differentiation and therefore myelin repair, reduces neuroinflammation in ALS model (inhibits NF-κB)	[51,52,53,54,55,56,57,58,59,60,61]
Curcumin	Dietary supplement	COVID-19	Protects and promotes repair of alveolar ATII cells in inflammatory lung injury model, increases Tregs, IL-10 and M2 macrophages in acute lung injury model, protects from cardiovascular injuries, reduces inflammation by inhibiting NF-κB	[62,63,64]
Ibuprofen	Pain relief	SCI	Promotes axon growth and motor function improvement in spinal cord injury models by RhoA inhibition	[65,66,67]
Indomethacin	Pain relief	SCI	Promotes axon growth in spinal cord lesion model by RhoA inhibition	[65,67]
Resolvin D1	-	Liver injury	Protects from IR-induced sterile liver inflammation by promoting M2 phenotype and efferocytosis in Kupffer cells, protects astrocytes and ameliorates cognitive impairment after TBI, inhibits inflammation and NF-κB signaling	[68,69,70,71,72]

**Table 5:** Drug repurposing candidates for inflammatory diseases targeting NF-κB signaling.

New Indication	Drug	Original Implication	Effect on NF-κB Signaling	Effect On Inflammation	References
MS	Imatinib mesylate	Cancer (CML, ALL, GIST, HES, CEL)	Inhibits IκB phosphorylation and DNA binding of NF-κB	Attenuates inflammation and enhances BBB integrity in EAE, Phase II clinical trial for MS	[183,184,185]
	Clemastine	Relief of allergy symptoms	Decreases NF-κB activity and TLR4 expression	Promotes oligodendrocyte differentiation and remyelination in EAE/MS, inhibits inflammation and microglial M1-like activation	[52,53,55,56,57,58,61]
	Ibudilast	Asthma, stroke	Inhibits NF-κB activity (possibly by preventing nuclear translocation)	Reduces inflammation in rats with chronic cerebral reperfusion and MS patients, Phase II clinical trial for MS	[186,187,188,189]
	Topotecan	Cancer (ovarian cancer, lung cancer, SCLC)	Inhibits IKKβ and thus IκBα degradation	Attenuates inflammation in EAE	[99]
RA	Ibrutinib	Cancer (MCL, CLL, WM)	Inhibits NF-κB nuclear translocation	Anti-inflammatory effects in models of RA, sepsis and diabetes	[190,191,192,193]
	Bortezomib	Cancer (MM, MCL)	Proteasome inhibitor, prevents degradation of IκBα	Anti-inflammatory effects in models of MS, RA, lupus erythematosus and colitis, promotes osteoblast activation and RA pathogenesis, Phase II clinical trial for RA	[194,195,196,197,198,199] [200,201]
	TDZ	Schizophrenia, psychosis	Inhibits IKKβ phosphorylation and IκBα degradation	Attenuates inflammation in endotoxemia model	[83]
	Dasatinib	Cancer (CML, ALL)	Inhibits phosphorylation of IKKα, p65/p100/p105 and c-Rel	Inhibits inflammation and bone erosion in CIA and human FLS, increases IL-10 in CIA	[122,202,203,204]
COVID-19	Dexamethasone	Inflammatory conditions (RA, asthma, allergies etc.)	Induces the expression of IκBα	Reduces mortality in later stage COVID-19 patients	[205,206,207,208]
	Anakinra	Relief of RA symptoms	Prevents activation of IL-1R	Reduces hyperinflammation and mortality and improves clinical signs of COVID-19	[209,210,211,212,213,214]

## Data Availability

Not applicable.

## References

[1] Sen R., Baltimore D. (1986). Multiple nuclear factors interact with the immunoglobulin enhancer sequences. Cell.

[2] Amiri K.I., Richmond A. (2005). Role of nuclear factor-kappa B in melanoma. Cancer Metastasis Rev..

[3] Chen F., Demers L.M., Shi X. (2002). Upstream signal transduction of NF-κB activation. Curr. Drug Targets-Inflamm. Allergy.

[4] Ben-Neriah Y. (2002). Regulatory functions of ubiquitination in the immune system. Nat. Immunol..

[5] Dorrington M.G., Fraser I.D.C. (2019). NF-κB Signaling in Macrophages: Dynamics, Crosstalk, and Signal Integration. Front. Immunol..

[6] Cai Z., Tchou-Wong K.-M., Rom W.N. (2011). NF-kappaB in Lung Tumorigenesis. Cancers.

[7] Imbert V., Peyron J.F. (2017). NF-κB in Hematological Malignancies. Biomedicines.

[8] Jiang L., Ren L., Zhang X., Chen H., Chen X., Lin C., Wang L., Hou N., Pan J., Zhou Z. (2019). Overexpression of PIMREG promotes breast cancer aggressiveness *via* constitutive activation of NF-κB signaling. EBioMedicine.

[9] Jiang L., Wu J., Yang Y., Liu L., Song L., Li J., Li M. (2012). Bmi-1 promotes the aggressiveness of glioma via activating the NF-kappaB/MMP-9 signaling pathway. BMC Cancer.

[10] Schön M., Wienrich B.G., Kneitz S., Sennefelder H., Amschler K., Vöhringer V., Weber O., Stiewe T., Ziegelbauer K., Schön M.P. (2008). KINK-1, a novel small-molecule inhibitor of IKKbeta, and the susceptibility of melanoma cells to antitumoral treatment. J. Natl. Cancer Inst..

[11] Taniguchi K., Karin M. (2018). NF-κB, inflammation, immunity and cancer: Coming of age. Nat. Rev. Immunol..

[12] Kumar A., Takada Y., Boriek A.M., Aggarwal B.B. (2004). Nuclear factor-kappaB: Its role in health and disease. J. Mol. Med..

[13] Liu T., Zhang L., Joo D., Sun S.-C. (2017). NF-κB signaling in inflammation. Signal Transduct. Target. Ther..

[14] Hayden M.S., West A.P., Ghosh S. (2006). NF-κB and the immune response. Oncogene.

[15] Nabel G.J., Verma I.M. (1993). Proposed NF-kappa B/I kappa B family nomenclature. Genes Dev..

[16] Mussbacher M., Salzmann M., Brostjan C., Hoesel B., Schoergenhofer C., Datler H., Hohensinner P., Basílio J., Petzelbauer P., Assinger A. (2019). Cell Type-Specific Roles of NF-κB Linking Inflammation and Thrombosis. Front. Immunol..

[17] Gurunathan S., Winkles J.A., Ghosh S., Hayden M.S. (2014). Regulation of fibroblast growth factor-inducible 14 (Fn14) expression levels via ligand-independent lysosomal degradation. J. Biol. Chem..

[18] Hoesel B., Schmid J.A. (2013). The complexity of NF-κB signaling in inflammation and cancer. Mol. Cancer.

[19] Wajant H., Siegmund D. (2019). TNFR1 and TNFR2 in the Control of the Life and Death Balance of Macrophages. Front. Cell Dev. Biol..

[20] Zhang H., Sun S.C. (2015). NF-κB in inflammation and renal diseases. Cell Biosci..

[21] Karin M., Delhase M. (2000). The I kappa B kinase (IKK) and NF-kappa B: Key elements of proinflammatory signalling. Semin. Immunol..

[22] Schröfelbauer B., Polley S., Behar M., Ghosh G., Hoffmann A. (2012). NEMO ensures signaling specificity of the pleiotropic IKKβ by directing its kinase activity toward IκBα. Mol. Cell.

[23] Sun S.C., Ley S.C. (2008). New insights into NF-kappaB regulation and function. Trends Immunol..

[24] Kanarek N., Ben-Neriah Y. (2012). Regulation of NF-κB by ubiquitination and degradation of the IκBs. Immunol. Rev..

[25] Beinke S., Ley S.C. (2004). Functions of NF-kappaB1 and NF-kappaB2 in immune cell biology. Biochem. J..

[26] Hayden M.S., Ghosh S. (2012). NF-κB, the first quarter-century: Remarkable progress and outstanding questions. Genes Dev..

[27] Sun S.C. (2011). Non-canonical NF-κB signaling pathway. Cell Res..

[28] Sun S.C., Liu Z.G. (2011). A special issue on NF-κB signaling and function. Cell Res..

[29] Cildir G., Low K.C., Tergaonkar V. (2016). Noncanonical NF-κB Signaling in Health and Disease. Trends Mol. Med..

[30] Senftleben U., Cao Y., Xiao G., Greten F.R., Krähn G., Bonizzi G., Chen Y., Hu Y., Fong A., Sun S.C. (2001). Activation by IKKalpha of a second, evolutionary conserved, NF-kappa B signaling pathway. Science.

[31] Xiao G., Harhaj E.W., Sun S.C. (2001). NF-kappaB-inducing kinase regulates the processing of NF-kappaB2 p100. Mol. Cell.

[32] Oeckinghaus A., Ghosh S. (2009). The NF-kappaB family of transcription factors and its regulation. Cold Spring Harb. Perspect. Biol..

[33] Prescott J.A., Mitchell J.P., Cook S.J. (2021). Inhibitory feedback control of NF-κB signalling in health and disease. Biochem. J..

[34] Yu H., Lin L., Zhang Z., Zhang H., Hu H. (2020). Targeting NF-κB pathway for the therapy of diseases: Mechanism and clinical study. Signal Transduct. Target. Ther..

[35] Zhao Z., Ukidve A., Kim J., Mitragotri S. (2020). Targeting Strategies for Tissue-Specific Drug Delivery. Cell.

[36] Alberti C., Pinciroli P., Valeri B., Ferri R., Ditto A., Umezawa K., Sensi M., Canevari S., Tomassetti A. (2012). Ligand-dependent EGFR activation induces the co-expression of IL-6 and PAI-1 via the NFkB pathway in advanced-stage epithelial ovarian cancer. Oncogene.

[37] Habib A.A., Chatterjee S., Park S.-K., Ratan R.R., Lefebvre S., Vartanian T. (2001). The epidermal growth factor receptor engages receptor interacting protein and nuclear factor-κB (NF-κB)-inducing kinase to activate NF-κB: Identification of a novel receptor-tyrosine kinase signalosome. J. Biol. Chem..

[38] Kato T., Delhase M., Hoffmann A., Karin M. (2003). CK2 is a C-terminal IκB kinase responsible for NF-κB activation during the UV response. Mol. Cell.

[39] Takada Y., Mukhopadhyay A., Kundu G.C., Mahabeleshwar G.H., Singh S., Aggarwal B.B. (2003). Hydrogen peroxide activates NF-κB through tyrosine phosphorylation of IκBα and serine phosphorylation of p65: Evidence for the involvement of IκBα kinase and Syk protein-tyrosine kinase. J. Biol. Chem..

[40] Ramadass V., Vaiyapuri T., Tergaonkar V. (2020). Small Molecule NF-κB Pathway Inhibitors in Clinic. Int. J. Mol. Sci..

[41] Gilmore T.D., Garbati M.R. (2011). Inhibition of NF-κB signaling as a strategy in disease therapy. Curr. Top. Microbiol. Immunol..

[42] Gilmore T.D., Herscovitch M. (2006). Inhibitors of NF-kappaB signaling: 785 and counting. Oncogene.

[43] Gupta S.C., Sundaram C., Reuter S., Aggarwal B.B. (2010). Inhibiting NF-κB activation by small molecules as a therapeutic strategy. Biochim. Biophys. Acta.

[44] Rainsford K.D. (1999). Profile and mechanisms of gastrointestinal and other side effects of nonsteroidal anti-inflammatory drugs (NSAIDs). Am. J. Med..

[45] Simon L.S. (2013). Nonsteroidal anti-inflammatory drugs and their risk: A story still in development. Arthritis Res. Ther..

[46] Nicolaides N.C., Pavlaki A.N., Alexandra M.A.M., Chrousos G.P. (2000). Glucocorticoid Therapy and Adrenal Suppression. Feingold.

[47] Oray M., Samra K.A., Ebrahimiadib N., Meese H., Foster C.S. (2016). Long-term side effects of glucocorticoids. Expert Opin. Drug Saf..

[48] (1999). TNF neutralization in MS: Results of a randomized, placebo-controlled multicenter study. Neurology.

[49] Fresegna D., Bullitta S., Musella A., Rizzo F.R., de Vito F., Guadalupi L., Caioli S., Balletta S., Sanna K., Dolcetti E. (2020). Re-Examining the Role of TNF in MS Pathogenesis and Therapy. Cells.

[50] Kemanetzoglou E., Andreadou E. (2017). CNS Demyelination with TNF-α Blockers. Curr. Neurol. Neurosci. Rep..

[51] Apolloni S., Fabbrizio P., Amadio S., Volonté C. (2016). Actions of the antihistaminergic clemastine on presymptomatic SOD1-G93A mice ameliorate ALS disease progression. J. Neuroinflamm..

[52] Cree B.A.C., Niu J., Hoi K.K., Zhao C., Caganap S.D., Henry R.G., Dao D.Q., Zollinger D.R., Mei F., Shen Y.-A.A. (2017). Clemastine rescues myelination defects and promotes functional recovery in hypoxic brain injury. Brain.

[53] Green A.J., Gelfand J.M., Cree B.A., Bevan C., Boscardin W.J., Mei F., Inman J., Arnow S., Devereux M., Abounasr A. (2017). Clemastine fumarate as a remyelinating therapy for multiple sclerosis (ReBUILD): A randomised, controlled, double-blind, crossover trial. Lancet.

[54] Leurs R., Church M.K., Taglialatela M. (2002). H1-antihistamines: Inverse agonism, anti-inflammatory actions and cardiac effects. Clin. Exp. Allergy.

[55] Li Z., He Y., Fan S., Sun B. (2015). Clemastine rescues behavioral changes and enhances remyelination in the cuprizone mouse model of demyelination. Neurosci. Bull..

[56] Liu J., Dupree J.L., Gacias M., Frawley R., Sikder T., Naik P., Casaccia P. (2016). Clemastine Enhances Myelination in the Prefrontal Cortex and Rescues Behavioral Changes in Socially Isolated Mice. J. Neurosci..

[57] Mei F., Fancy S.P.J., Shen Y.A., Niu J., Zhao C., Presley B., Miao E., Lee S., Mayoral S.R., Redmond S.A. (2014). Micropillar arrays as a high-throughput screening platform for therapeutics in multiple sclerosis. Nat. Med..

[58] Mei F., Lehmann-Horn K., Shen Y.-A.A., Rankin K.A., Stebbins K.J., Lorrain D.S., Pekarek K., Sagan S.A., Xiao L., Teuscher C. (2016). Accelerated remyelination during inflammatory demyelination prevents axonal loss and improves functional recovery. Elife.

[59] Su W.J., Zhang T., Jiang C.L., Wang W. (2018). Clemastine Alleviates Depressive-Like Behavior Through Reversing the Imbalance of Microglia-Related Pro-inflammatory State in Mouse Hippocampus. Front. Cell. Neurosci..

[60] Yuan X., Juan Z., Zhang R., Sun X., Yan R., Yue F., Huang Y., Yu J., Xia X. (2020). Clemastine Fumarate Protects Against Myocardial Ischemia Reperfusion Injury by Activating the TLR4/PI3K/Akt Signaling Pathway. Front. Pharmacol..

[61] Zada D., Tovin A., Lerer-Goldshtein T., Appelbaum L. (2016). Pharmacological treatment and BBB-targeted genetic therapy for MCT8-dependent hypomyelination in zebrafish. Dis. Models Mech..

[62] Almatroodi S.A., Alrumaihi F., Alsahli M.A., Alhommrani M.F., Khan A., Rahmani A.H. (2020). Curcumin, an Active Constituent of Turmeric Spice: Implication in the Prevention of Lung Injury Induced by Benzo(a) Pyrene (BaP) in Rats. Molecules.

[63] Chai Y.-S., Chen Y.-Q., Lin S.-H., Xie K., Wang C.-J., Yang Y.-Z., Xu F. (2020). Curcumin regulates the differentiation of naïve CD4+T cells and activates IL-10 immune modulation against acute lung injury in mice. Biomed. Pharmacother..

[64] Soni V.K., Mehta A., Ratre Y.K., Tiwari A.K., Amit A., Singh R.P., Sonkar S.C., Chaturvedi N., Shukla D., Vishvakarma N.K. (2020). Curcumin, a traditional spice component, can hold the promise against COVID-19?. Eur. J. Pharmacol..

[65] Fu Q., Hue J., Li S. (2007). Nonsteroidal Anti-Inflammatory Drugs Promote Axon Regeneration via RhoA Inhibition. J. Neurosci..

[66] Kopp M.A., Liebscher T., Watzlawick R., Martus P., Laufer S., Blex C., Schindler R., Jungehulsing G.J., Knüppel S., Kreutzträger M. (2016). SCISSOR-Spinal Cord Injury Study on Small molecule-derived Rho inhibition: A clinical study protocol. BMJ Open.

[67] Lambrechts M.J., Cook J.L. (2021). Nonsteroidal Anti-Inflammatory Drugs and Their Neuroprotective Role After an Acute Spinal Cord Injury: A Systematic Review of Animal Models. Glob. Spine J..

[68] Bento A.F., Claudino R.F., Dutra R.C., Marcon R., Calixto J.B. (2011). Omega-3 fatty acid-derived mediators 17 (R)-hydroxy docosahexaenoic acid, aspirin-triggered resolvin D1 and resolvin D2 prevent experimental colitis in mice. J. Immunol..

[69] Kang J.-W., Lee S.-M. (2016). Resolvin D1 protects the liver from ischemia/reperfusion injury by enhancing M2 macrophage polarization and efferocytosis. Biochim. Biophys. Acta (BBA)-Mol. Cell Biol. Lipids.

[70] Ren Y.-Z., Zhang B.-Z., Zhao X.-J., Zhang Z.-Y. (2020). Resolvin D1 ameliorates cognitive impairment following traumatic brain injury via protecting astrocytic mitochondria. J. Neurochem..

[71] Wang B., Gong X., Wan J.-y., Zhang L., Zhang Z., Li H.-z., Min S. (2011). Resolvin D1 protects mice from LPS-induced acute lung injury. Pulm. Pharmacol. Ther..

[72] Wei C., Guo S., Liu W., Jin F., Wei B., Fan H., Su H., Liu J., Zhang N., Fang D. (2021). Resolvin D1 ameliorates Inflammation-Mediated Blood-Brain Barrier Disruption after Subarachnoid Hemorrhage in rats by Modulating A20 and NLRP3 Inflammasome. Front. Pharmacol..

[73] Jang D., Lee S., Lee J., Kim K., Lee D. (2016). Inferring new drug indications using the complementarity between clinical disease signatures and drug effects. J. Biomed. Inform..

[74] Chong C.R., Sullivan D.J. (2007). New uses for old drugs. Nature.

[75] Nosengo N. (2016). Can you teach old drugs new tricks?. Nature.

[76] Low Z.Y., Farouk I.A., Lal S.K. (2020). Drug Repositioning: New Approaches and Future Prospects for Life-Debilitating Diseases and the COVID-19 Pandemic Outbreak. Viruses.

[77] Prakash A.V., Park J.W., Seong J.-W., Kang T.J. (2020). Repositioned Drugs for Inflammatory Diseases such as Sepsis, Asthma, and Atopic Dermatitis. Biomol. Ther..

[78] Pushpakom S., Iorio F., Eyers P.A., Escott K.J., Hopper S., Wells A., Doig A., Guilliams T., Latimer J., McNamee C. (2019). Drug repurposing: Progress, challenges and recommendations. Nat. Rev. Drug Discov..

[79] Ghofrani H.A., Osterloh I.H., Grimminger F. (2006). Sildenafil: From angina to erectile dysfunction to pulmonary hypertension and beyond. Nat. Rev. Drug Discov..

[80] Dudley J.T., Deshpande T., Butte A.J. (2011). Exploiting drug-disease relationships for computational drug repositioning. Brief. Bioinform..

[81] Sam E., Athri P. (2017). Web-based drug repurposing tools: A survey. Brief. Bioinform..

[82] Xue H., Li J., Xie H., Wang Y. (2018). Review of Drug Repositioning Approaches and Resources. Int. J. Biol. Sci..

[83] Baig M.S., Roy A., Saqib U., Rajpoot S., Srivastava M., Naim A., Liu D., Saluja R., Faisal S.M., Pan Q. (2018). Repurposing Thioridazine (TDZ) as an anti-inflammatory agent. Sci. Rep..

[84] Feinberg S.M., Saadabadi A.F.K. (2022). Thioridazine. https://www.ncbi.nlm.nih.gov/books/NBK459140/.

[85] Anthony N.G., Baiget J., Berretta G., Boyd M., Breen D., Edwards J., Gamble C., Gray A.I., Harvey A.L., Hatziieremia S. (2017). Inhibitory Kappa B Kinase α (IKKα) Inhibitors That Recapitulate Their Selectivity in Cells against Isoform-Related Biomarkers. J. Med. Chem..

[86] Llona-Minguez S., Baiget J., Mackay S.P. (2013). Small-molecule inhibitors of IκB kinase (IKK) and IKK-related kinases. Pharm. Pat. Anal..

[87] Zhou Y., Cui C., Ma X., Luo W., Zheng S.G., Qiu W. (2020). Nuclear Factor κB (NF-κB)–Mediated Inflammation in Multiple Sclerosis. Front. Immunol..

[88] Corsello S.M., Bittker J.A., Liu Z., Gould J., McCarren P., Hirschman J.E., Johnston S.E., Vrcic A., Wong B., Khan M. (2017). The Drug Repurposing Hub: A next-generation drug library and information resource. Nat. Med..

[89] Avram S., Bologa C.G., Holmes J., Bocci G., Wilson T.B., Nguyen D.-T., Curpan R., Halip L., Bora A., Yang J.J. (2020). DrugCentral 2021 supports drug discovery and repositioning. Nucleic Acids Res..

[90] Gaziano L., Giambartolomei C., Pereira A.C., Gaulton A., Posner D.C., Swanson S.A., Ho Y.-L., Iyengar S.K., Kosik N.M., Vujkovic M. (2021). Actionable druggable genome-wide Mendelian randomization identifies repurposing opportunities for COVID-19. Nat. Med..

[91] Mendez D., Gaulton A., Bento A.P., Chambers J., de Veij M., Félix E., Magariños M.P., Mosquera J.F., Mutowo P., Nowotka M. (2019). ChEMBL: Towards direct deposition of bioassay data. Nucleic Acids Res..

[92] Pantziarka P., Vandeborne L., Bouche G. (2021). A Database of Drug Repurposing Clinical Trials in Oncology. Front. Pharmacol..

[93] Brown A.S., Patel C.J. (2017). A standard database for drug repositioning. Sci. Data.

[94] Janes J., Young M.E., Chen E., Rogers N.H., Burgstaller-Muehlbacher S., Hughes L.D., Love M.S., Hull M.V., Kuhen K.L., Woods A.K. (2018). The ReFRAME library as a comprehensive drug repurposing library and its application to the treatment of cryptosporidiosis. Proc. Natl. Acad. Sci. USA.

[95] Irwin J.J., Shoichet B.K. (2005). ZINC—A Free Database of Commercially Available Compounds for Virtual Screening. J. Chem. Inf. Model..

[96] (2020). COVID-19 Drug Repurposing Database. https://www.excelra.com/covid-19-drug-repurposing-database/.

[97] Drug Repurposing Online. https://drugrepurposing.info/.

[98] von Eichborn J., Murgueitio M.S., Dunkel M., Koerner S., Bourne P.E., Preissner R. (2010). PROMISCUOUS: A database for network-based drug-repositioning. Nucleic Acids Res..

[99] Zeng X., Zhu S., Lu W., Liu Z., Huang J., Zhou Y., Fang J., Huang Y., Guo H., Li L. (2020). Target identification among known drugs by deep learning from heterogeneous networks. Chem. Sci..

[100] Zeng X., Zhu S., Hou Y., Zhang P., Li L., Li J., Huang L.F., Lewis S.J., Nussinov R., Cheng F. (2020). Network-based prediction of drug–target interactions using an arbitrary-order proximity embedded deep forest. Bioinformatics.

[101] Luo H., Wang J., Li M., Luo J., Peng X., Wu F.X., Pan Y. (2016). Drug repositioning based on comprehensive similarity measures and Bi-Random walk algorithm. Bioinformatics.

[102] Kuusisto F., Steill J., Kuang Z., Thomson J., Page D., Stewart R. (2017). A Simple Text Mining Approach for Ranking Pairwise Associations in Biomedical Applications. AMIA Jt. Summits Transl. Sci. Proc..

[103] Papanikolaou N., Pavlopoulos G.A., Theodosiou T., Vizirianakis I.S., Iliopoulos I. (2016). DrugQuest-a text mining workflow for drug association discovery. BMC Bioinform..

[104] Chen B., Ding Y., Wild D.J. (2012). Assessing drug target association using semantic linked data. PLoS Comput. Biol..

[105] Luu T. (2020). Pharmacophore-Guided Virtual Screening for Drug Repurposing. https://blogs.3ds.com/biovia/pharmacophore-guided-virtual-screening-for-drug-repurposing/.

[106] Hodos R.A., Kidd B.A., Shameer K., Readhead B.P., Dudley J.T. (2016). In silico methods for drug repurposing and pharmacology. Wiley Interdiscip. Rev. Syst. Biol. Med..

[107] Ali E.M.H., Abdel-Maksoud M.S., Hassan R.M., Mersal K.I., Ammar U.M., Se-In C., He-Soo H., Kim H.K., Lee A., Lee K.T. (2021). Design, synthesis and anti-inflammatory activity of imidazol-5-yl pyridine derivatives as p38α/MAPK14 inhibitor. Bioorg. Med. Chem..

[108] Kanan T., Kanan D., Erol I., Yazdi S., Stein M., Durdagi S. (2019). Targeting the NF-κB/IκBα complex via fragment-based E-Pharmacophore virtual screening and binary QSAR models. J. Mol. Graph. Model..

[109] Balaramnavar V.M., Ahmad K., Saeed M., Ahmad I., Kamal M., Jawed T. (2020). Pharmacophore-based approaches in the rational repurposing technique for FDA approved drugs targeting SARS-CoV-2 Mpro. RSC Adv..

[110] Rampogu S., Lee K.W. (2021). Pharmacophore Modelling-Based Drug Repurposing Approaches for SARS-CoV-2 Therapeutics. Front. Chem..

[111] Sundaresan L., Giri S., Singh H., Chatterjee S. (2021). Repurposing of thalidomide and its derivatives for the treatment of SARS-CoV-2 infections: Hints on molecular action. Br. J. Clin. Pharmacol..

[112] Rajan S., Satish S., Shankar K., Pandeti S., Varshney S., Srivastava A., Kumar D., Gupta A., Gupta S., Choudhary R. (2018). Aegeline inspired synthesis of novel β3-AR agonist improves insulin sensitivity in vitro and in vivo models of insulin resistance. Metabolism.

[113] Chen C., Qi F., Shi K., Li Y., Li J., Chen Y., Pan J., Zhou T., Lin X., Zhang J. (2020). Thalidomide combined with low-dose short-term glucocorticoid in the treatment of critical Coronavirus Disease 2019. Clin. Transl. Med..

[114] Li Y., Shi K., Qi F., Yu Z., Chen C., Pan J., Wu G., Chen Y., Li J., Chen Y. (2021). Thalidomide combined with short-term low-dose glucocorticoid therapy for the treatment of severe COVID-19: A case-series study. Int. J. Infect. Dis..

[115] Bertolini F., Sukhatme V.P., Bouche G. (2015). Drug repurposing in oncology—Patient and health systems opportunities. Nat. Rev. Clin. Oncol..

[116] Ozanne J., Prescott A.R., Clark K. (2015). The clinically approved drugs dasatinib and bosutinib induce anti-inflammatory macrophages by inhibiting the salt-inducible kinases. Biochem. J..

[117] Pizzorno A., Padey B., Terrier O., Rosa-Calatrava M. (2019). Drug Repurposing Approaches for the Treatment of Influenza Viral Infection: Reviving Old Drugs to Fight against a Long-Lived Enemy. Front. Immunol..

[118] D’Aura Swanson C., Paniagua R.T., Lindstrom T.M., Robinson W.H. (2009). Tyrosine kinases as targets for the treatment of rheumatoid arthritis. Nat. Rev. Rheumatol..

[119] Keating G.M. (2017). Dasatinib: A review in chronic myeloid leukaemia and Ph+ acute lymphoblastic leukaemia. Drugs.

[120] Meeker N.D., Trede N.S. (2008). Immunology and zebrafish: Spawning new models of human disease. Dev. Comp. Immunol..

[121] Traver D., Herbomel P., Patton E., Murphey R.D., Yoder J., Litman G., Catic A., Amemiya C., Zon L., Trede N. (2003). The zebrafish as a model organism to study development of the immune system. Adv. Immunol..

[122] Guo K., Bu X., Yang C., Cao X., Bian H., Zhu Q., Zhu J., Zhang D. (2019). Treatment Effects of the Second-Generation Tyrosine Kinase Inhibitor Dasatinib on Autoimmune Arthritis. Front. Immunol..

[123] Hekim C., Ilander M., Yan J., Michaud E., Smykla R., Vähä-Koskela M., Savola P., Tähtinen S., Saikko L., Hemminki A. (2017). Dasatinib changes immune cell profiles concomitant with reduced tumor growth in several murine solid tumor models. Cancer Immunol. Res..

[124] Day E., Waters B., Spiegel K., Alnadaf T., Manley P.W., Buchdunger E., Walker C., Jarai G. (2008). Inhibition of collagen-induced discoidin domain receptor 1 and 2 activation by imatinib, nilotinib and dasatinib. Eur. J. Pharmacol..

[125] Korashy H.M., Rahman A.M., Kassem M.G. (2014). Dasatinib. Profiles Drug Subst. Excip. Relat. Methodol..

[126] Hay M., Thomas D.W., Craighead J.L., Economides C., Rosenthal J. (2014). Clinical development success rates for investigational drugs. Nat. Biotechnol..

[127] Nierode G., Kwon P.S., Dordick J.S., Kwon S.J. (2016). Cell-Based Assay Design for High-Content Screening of Drug Candidates. J. Microbiol. Biotechnol..

[128] Emery B. (2010). Regulation of oligodendrocyte differentiation and myelination. Science.

[129] Xie Y., Meijer A.H., Schaaf M.J.M. (2020). Modeling Inflammation in Zebrafish for the Development of Anti-inflammatory Drugs. Front. Cell Dev. Biol..

[130] Hall C.J., Wicker S.M., Chien A.-T., Tromp A., Lawrence L.M., Sun X., Krissansen G.W., Crosier K.E., Crosier P.S. (2014). Repositioning drugs for inflammatory disease–fishing for new anti-inflammatory agents. Dis. Models Mech..

[131] Herbomel P., Thisse B., Thisse C. (1999). Ontogeny and behaviour of early macrophages in the zebrafish embryo. Development.

[132] Hermann A.C., Millard P.J., Blake S.L., Kim C.H. (2004). Development of a respiratory burst assay using zebrafish kidneys and embryos. J. Immunol. Methods.

[133] Lam S.H., Chua H.L., Gong Z., Lam T.J., Sin Y.M. (2004). Development and maturation of the immune system in zebrafish, Danio rerio: A gene expression profiling, in situ hybridization and immunological study. Dev. Comp. Immunol..

[134] Page D.M., Wittamer V., Bertrand J.Y., Lewis K.L., Pratt D.N., Delgado N., Schale S.E., McGue C., Jacobsen B.H., Doty A. (2013). An evolutionarily conserved program of B-cell development and activation in zebrafish. Blood.

[135] White D.T., Saxena M.T., Mumm J.S. (2019). Let’s get small (and smaller): Combining zebrafish and nanomedicine to advance neuroregenerative therapeutics. Adv. Drug Deliv. Rev..

[136] Rihel J., Prober D.A., Arvanites A., Lam K., Zimmerman S., Jang S., Haggarty S.J., Kokel D., Rubin L.L., Peterson R.T. (2010). Zebrafish behavioral profiling links drugs to biological targets and rest/wake regulation. Science.

[137] Ghasemi N., Razavi S., Nikzad E. (2017). Multiple Sclerosis: Pathogenesis, Symptoms, Diagnoses and Cell-Based Therapy. Cell J..

[138] Dendrou C.A., Fugger L., Friese M.A. (2015). Immunopathology of multiple sclerosis. Nat. Rev. Immunol..

[139] Beecham A.H., Patsopoulos N.A., Xifara D.K., Davis M.F., Kemppinen A., Cotsapas C., Shah T.S., Spencer C., Booth D., Goris A. (2013). Analysis of immune-related loci identifies 48 new susceptibility variants for multiple sclerosis. Nat. Genet.

[140] Hussman J.P., Beecham A.H., Schmidt M., Martin E.R., McCauley J.L., Vance J.M., Haines J.L., Pericak-Vance M.A. (2016). GWAS analysis implicates NF-κB-mediated induction of inflammatory T cells in multiple sclerosis. Genes Immun..

[141] Miterski B., Böhringer S., Klein W., Sindern E., Haupts M., Schimrigk S., Epplen J.T. (2002). Inhibitors in the NFκB cascade comprise prime candidate genes predisposing to multiple sclerosis, especially in selected combinations. Genes Immun..

[142] Oh H., Ghosh S. (2013). NF-κB: Roles and regulation in different CD4(+) T-cell subsets. Immunol. Rev..

[143] Ruan Q., Chen Y.H. (2012). Nuclear factor-κB in immunity and inflammation: The Treg and Th17 connection. Adv. Exp. Med. Biol..

[144] Collins T., Read M.A., Neish A.S., Whitley M.Z., Thanos D., Maniatis T. (1995). Transcriptional regulation of endothelial cell adhesion molecules: NF-kappa B and cytokine-inducible enhancers. FASEB J..

[145] Nomura T., Abe Y., Kamada H., Shibata H., Kayamuro H., Inoue M., Kawara T., Arita S., Furuya T., Yamashita T. (2011). Therapeutic effect of PEGylated TNFR1-selective antagonistic mutant TNF in experimental autoimmune encephalomyelitis mice. J. Control Release.

[146] Petrasch S. (1995). Follicular dendritic cells in malignant lymphomas. Curr. Top. Microbiol. Immunol..

[147] van Doorn R., Pinheiro M.A.L., Kooij G., Lakeman K., Hof B.v., van der Pol S.M., Geerts D., van Horssen J., van der Valk P., van der Kam E. (2012). Sphingosine 1-phosphate receptor 5 mediates the immune quiescence of the human brain endothelial barrier. J. Neuroinflamm..

[148] Chen G., Hardy K., Pagler E., Ma L., Lee S., Gerondakis S., Daley S., Shannon M.F. (2011). The NF-κB transcription factor c-Rel is required for Th17 effector cell development in experimental autoimmune encephalomyelitis. J. Immunol..

[149] Greve B., Weissert R., Hamdi N., Bettelli E., Sobel R.A., Coyle A., Kuchroo V.K., Rajewsky K., Schmidt-Supprian M. (2007). IκB Kinase 2/β Deficiency Controls Expansion of Autoreactive T Cells and Suppresses Experimental Autoimmune Encephalomyelitis. J. Immunol..

[150] Hilliard B., Samoilova E.B., Liu T.-S.T., Rostami A., Chen Y. (1999). Experimental Autoimmune Encephalomyelitis in NF-κB- Deficient Mice: Roles of NF-κB in the Activation and Differentiation of Autoreactive T Cells. J. Immunol..

[151] Hilliard B.A., Mason N., Xu L., Sun J., Lamhamedi-Cherradi S.E., Liou H.C., Hunter C., Chen Y.H. (2002). Critical roles of c-Rel in autoimmune inflammation and helper T cell differentiation. J. Clin. Investig..

[152] Jin W., Zhou X.F., Yu J., Cheng X., Sun S.C. (2009). Regulation of Th17 cell differentiation and EAE induction by MAP3K NIK. Blood.

[153] Li Y., Wang H., Zhou X., Xie X., Chen X., Jie Z., Zou Q., Hu H., Zhu L., Cheng X. (2016). Cell intrinsic role of NF-κB-inducing kinase in regulating T cell-mediated immune and autoimmune responses. Sci. Rep..

[154] Yue Y., Stone S., Lin W. (2018). Role of nuclear factor κB in multiple sclerosis and experimental autoimmune encephalomyelitis. Neural Regen. Res..

[155] Constantinescu C.S., Farooqi N., O’Brien K., Gran B. (2011). Experimental autoimmune encephalomyelitis (EAE) as a model for multiple sclerosis (MS). Br. J. Pharmacol..

[156] Hayes C.E., Spanier J.A., Watson R.R., Killgore W.D.S. (2017). Chapter 10-Multiple Sclerosis in Women: Vitamin D and Estrogen Synergy for Autoimmune T-Cell Regulation and Demyelinating Disease Prevention. Nutrition and Lifestyle in Neurological Autoimmune Diseases.

[157] Phelan J. (2016). Generating EAE Mouse Models of Multiple Sclerosis. https://www.taconic.com/taconic-insights/neuroscience/eae-mouse-models-of-multiple-sclerosis.html.

[158] Blank T., Prinz M. (2014). NF-κB signaling regulates myelination in the CNS. Front. Mol. Neurosci..

[159] Leibowitz S.M., Yan J. (2016). NF-κB Pathways in the Pathogenesis of Multiple Sclerosis and the Therapeutic Implications. Front. Mol. Neurosci..

[160] Ellrichmann G., Thöne J., Lee D.-H., Rupec R.A., Gold R., Linker R.A. (2012). Constitutive activity of NF-kappa B in myeloid cells drives pathogenicity of monocytes and macrophages during autoimmune neuroinflammation. J. Neuroinflamm..

[161] Hao W., Decker Y., Schnöder L., Schottek A., Li D., Menger M.D., Fassbender K., Liu Y. (2016). Deficiency of IκB Kinase β in Myeloid Cells Reduces Severity of Experimental Autoimmune Encephalomyelitis. Am. J. Pathol..

[162] Lee M.J., Bing S.J., Choi J., Jang M., Lee G., Lee H., Chang B.S., Jee Y., Lee S.J., Cho I.-H. (2016). IKKβ-mediated inflammatory myeloid cell activation exacerbates experimental autoimmune encephalomyelitis by potentiating Th1/Th17 cell activation and compromising blood brain barrier. Mol. Neurodegener..

[163] Goldmann T., Wieghofer P., Müller P.F., Wolf Y., Varol D., Yona S., Brendecke S.M., Kierdorf K., Staszewski O., Datta M. (2013). A new type of microglia gene targeting shows TAK1 to be pivotal in CNS autoimmune inflammation. Nat. Neurosci..

[164] Takahashi K., Prinz M., Stagi M., Chechneva O., Neumann H. (2007). TREM2-transduced myeloid precursors mediate nervous tissue debris clearance and facilitate recovery in an animal model of multiple sclerosis. PLoS Med..

[165] Brüstle A., Brenner D., Knobbe C.B., Lang P.A., Virtanen C., Hershenfield B.M., Reardon C., Lacher S.M., Ruland J., Ohashi P.S. (2012). The NF-κB regulator MALT1 determines the encephalitogenic potential of Th17 cells. J. Clin. Investig..

[166] Mc Guire C., Wieghofer P., Elton L., Muylaert D., Prinz M., Beyaert R., van Loo G. (2013). Paracaspase MALT1 deficiency protects mice from autoimmune-mediated demyelination. J. Immunol..

[167] Molinero L.L., Cubre A., Mora-Solano C., Wang Y., Alegre M.L. (2012). T cell receptor/CARMA1/NF-κB signaling controls T-helper (Th) 17 differentiation. Proc. Natl. Acad. Sci. USA.

[168] Hofmann J., Mair F., Greter M., Schmidt-Supprian M., Becher B. (2011). NIK signaling in dendritic cells but not in T cells is required for the development of effector T cells and cell-mediated immune responses. J. Exp. Med..

[169] Voet S., Guire C.M., Hagemeyer N., Martens A., Schroeder A., Wieghofer P., Daems C., Staszewski O., Walle L.V., Jordao M.J.C. (2018). A20 critically controls microglia activation and inhibits inflammasome-dependent neuroinflammation. Nat. Commun..

[170] Brambilla R., Dvoriantchikova G., Barakat D., Ivanov D., Bethea J.R., Shestopalov V.I. (2012). Transgenic inhibition of astroglial NF-κB protects from optic nerve damage and retinal ganglion cell loss in experimental optic neuritis. J. Neuroinflamm..

[171] Brambilla R., Morton P.D., Ashbaugh J.J., Karmally S., Lambertsen K.L., Bethea J.R. (2014). Astrocytes play a key role in EAE pathophysiology by orchestrating in the CNS the inflammatory response of resident and peripheral immune cells and by suppressing remyelination. Glia.

[172] Brambilla R., Persaud T., Hu X., Karmally S., Shestopalov V.I., Dvoriantchikova G., Ivanov D., Nathanson L., Barnum S.R., Bethea J.R. (2009). Transgenic Inhibition of Astroglial NF-κB Improves Functional Outcome in Experimental Autoimmune Encephalomyelitis by Suppressing Chronic Central Nervous System Inflammation. J. Immunol..

[173] Raasch J., Zeller N., van Loo G., Merkler D., Mildner A., Erny D., Knobeloch K.P., Bethea J.R., Waisman A., Knust M. (2011). IkappaB kinase 2 determines oligodendrocyte loss by non-cell-autonomous activation of NF-kappaB in the central nervous system. Brain.

[174] Brambilla R. (2019). The contribution of astrocytes to the neuroinflammatory response in multiple sclerosis and experimental autoimmune encephalomyelitis. Acta Neuropathol..

[175] Wang X., Deckert M., Xuan N.T., Nishanth G., Just S., Waisman A., Naumann M., Schlüter D. (2013). Astrocytic A20 ameliorates experimental autoimmune encephalomyelitis by inhibiting NF-κB-and STAT1-dependent chemokine production in astrocytes. Acta Neuropathol..

[176] Stone S., Jamison S., Yue Y., Durose W., Schmidt-Ullrich R., Lin W. (2017). NF-κB Activation Protects Oligodendrocytes against Inflammation. J. Neurosci..

[177] Emmanouil M., Taoufik E., Tseveleki V., Vamvakas S.-S., Tselios T., Karin M., Lassmann H., Probert L. (2009). Neuronal I kappa B kinase beta protects mice from autoimmune encephalomyelitis by mediating neuroprotective and immunosuppressive effects in the central nervous system. J. Immunol..

[178] Lee D.H., Kubera K., Rosenthal B., Kaltschmidt B., Kaltschmidt C., Gold R., Linker R.A. (2012). Neuronal NF-κB ablation does not influence neuro-axonal degeneration in experimental autoimmune demyelination. J. Neuroimmunol..

[179] van Loo G., de Lorenzi R., Schmidt H., Huth M., Mildner A., Schmidt-Supprian M., Lassmann H., Prinz M.R., Pasparakis M. (2006). Inhibition of transcription factor NF-κB in the central nervous system ameliorates autoimmune encephalomyelitis in mice. Nat. Immunol..

[180] Bonetti B., Stegagno C., Cannella B., Rizzuto N., Moretto G., Raine C.S. (1999). Activation of NF-κB and c-jun transcription factors in multiple sclerosis lesions: Implications for oligodendrocyte pathology. Am. J. Pathol..

[181] Gupta A.S., Biswas D.D., Brown L.S.N., Mockenhaupt K., Marone M., Hoskins A., Siebenlist U., Kordula T. (2019). A detrimental role of RelB in mature oligodendrocytes during experimental acute encephalomyelitis. J. Neuroinflamm..

[182] Mc Guire C., Prinz M., Beyaert R., van Loo G. (2013). Nuclear factor kappa B (NF-κB) in multiple sclerosis pathology. Trends Mol. Med..

[183] Bärnthaler T., Jandl K., Sill H., Uhl B., Schreiber Y., Grill M., Thomas D., Schicho R., Marsche G., Frank S. (2019). Imatinib stimulates prostaglandin E(2) and attenuates cytokine release via EP4 receptor activation. J. Allergy Clin. Immunol..

[184] Ciarcia R., Vitiello M.T., Galdiero M., Pacilio C., Iovane V., d’Angelo D., Pagnini D., Caparrotti G., Conti D., Tomei V. (2012). Imatinib treatment inhibit IL-6, IL-8, NF-KB and AP-1 production and modulate intracellular calcium in CML patients. J. Cell Physiol..

[185] Wolf A.M., Wolf D., Rumpold H., Ludwiczek S., Enrich B., Gastl G., Weiss G., Tilg H. (2005). The kinase inhibitor imatinib mesylate inhibits TNF-α production in vitro and prevents TNF-dependent acute hepatic inflammation. Proc. Natl. Acad. Sci. USA.

[186] Feng J., Misu T., Fujihara K., Sakoda S., Nakatsuji Y., Fukaura H., Kikuchi S., Tashiro K., Suzumura A., Ishii N. (2004). Ibudilast, a nonselective phosphodiesterase inhibitor, regulates Th1/Th2 balance and NKT cell subset in multiple sclerosis. Mult. Scler. J..

[187] Ghazi A., Abood S.H., Alaqouli H., Hadi N., Janabi S.A.M.A.M. (2019). Ibudilast and octreotide can ameliorate acute pancreatitis via downregulation of the inflammatory cytokines and Nuclear Factor- Kappa B expression. Ann. Trop. Med. Public Health.

[188] Kiebala M., Maggirwar S.B. (2011). Ibudilast, a pharmacologic phosphodiesterase inhibitor, prevents human immunodeficiency virus-1 Tat-mediated activation of microglial cells. PLoS ONE.

[189] Wakita H., Tomimoto H., Akiguchi I., Lin J.-X., Ihara M., Ohtani R., Shibata M. (2003). Ibudilast, a phosphodiesterase inhibitor, protects against white matter damage under chronic cerebral hypoperfusion in the rat. Brain Res..

[190] Chang B.Y., Huang M.M., Francesco M., Chen J., Sokolove J., Magadala P., Robinson W.H., Buggy J.J. (2011). The Bruton tyrosine kinase inhibitor PCI-32765 ameliorates autoimmune arthritis by inhibition of multiple effector cells. Arthritis Res. Ther..

[191] O’Riordan C.E., Purvis G.S.D., Collotta D., Chiazza F., Wissuwa B., al Zoubi S., Stiehler L., Martin L., Coldewey S.M., Collino M. (2019). Bruton’s Tyrosine Kinase Inhibition Attenuates the Cardiac Dysfunction Caused by Cecal Ligation and Puncture in Mice. Front. Immunol..

[192] Pan Z., Scheerens H., Li S.-J., Schultz B.E., Sprengeler P.A., Burrill L.C., Mendonca R.V., Sweeney M.D., Scott K.C.K., Grothaus P.G. (2007). Discovery of Selective Irreversible Inhibitors for Bruton’s Tyrosine Kinase. ChemMedChem.

[193] Purvis G.S.D., Collino M., Aranda-Tavio H., Chiazza F., O’Riordan C.E., Zeboudj L., Mohammad S., Collotta D., Verta R., Guisot N.E.S. (2020). Inhibition of Bruton’s TK regulates macrophage NF-κB and NLRP3 inflammasome activation in metabolic inflammation. Br. J. Pharmacol..

[194] Basler M., Dajee M., Moll C., Groettrup M., Kirk C.J. (2010). Prevention of experimental colitis by a selective inhibitor of the immunoproteasome. J. Immunol..

[195] Fissolo N., Kraus M., Reich M., Ayturan M., Overkleeft H., Driessen C., Weissert R. (2008). Dual inhibition of proteasomal and lysosomal proteolysis ameliorates autoimmune central nervous system inflammation. Eur. J. Immunol..

[196] Herrington F.D., Carmody R.J., Goodyear C.S. (2016). Modulation of NF-κB Signaling as a Therapeutic Target in Autoimmunity. J. Biomol. Screen..

[197] Lassoued S., Moyano C., Beldjerd M., Pauly P., Lassoued D., Billey T. (2019). Bortezomib improved the joint manifestations of rheumatoid arthritis in three patients. Jt. Bone Spine.

[198] Liu J., Li J., Chen M., Kuang L. (2016). Bortezomib followed by autologous stem cell transplantation in a patient with rheumatoid arthritis: A case report and review of the literature. Medicine.

[199] Schmidt N., Gonzalez E., Visekruna A., Kühl A.A., Loddenkemper C., Mollenkopf H., Kaufmann S.H., Steinhoff U., Joeris T. (2010). Targeting the proteasome: Partial inhibition of the proteasome by bortezomib or deletion of the immunosubunit LMP7 attenuates experimental colitis. Gut.

[200] Lee S.W., Kim J.H., Park Y.B., Lee S.K. (2009). Bortezomib attenuates murine collagen-induced arthritis. Ann. Rheum. Dis..

[201] Verbrugge S.E., Scheper R.J., Lems W.F., de Gruijl T.D., Jansen G. (2015). Proteasome inhibitors as experimental therapeutics of autoimmune diseases. Arthritis Res. Ther..

[202] Cooper M.J., Cox N.J., Zimmerman E.I., Dewar B.J., Duncan J.S., Whittle M.C., Nguyen T.A., Jones L.S., Roy S.G., Smalley D.M. (2013). Application of multiplexed kinase inhibitor beads to study kinome adaptations in drug-resistant leukemia. PLoS ONE.

[203] Fei F., Yu Y., Schmitt A., Chen J., Chen B., Rojewski M., Ringhoffer M., von Harsdorf S., Greiner J., Goetz M. (2007). Tyrosine Kinase Inhibitors Dasatinib, Nilotinib and Imatinib Have an Impact on Both CD8+ T Lymphocytes and CD4+CD25+FoxP3+ Regulatory T Cells by Downregulation of the NF-κB Pathway. Blood.

[204] Fei F., Yu Y., Schmitt A., Rojewski M.T., Chen B., Greiner J., Götz M., Bunjes D., Schmitt M. (2010). Effects of nilotinib on regulatory T cells: The dose matters. Mol. Cancer.

[205] Horby P., Lim W.S., Emberson J.R., Mafham M., Bell J.L., Linsell L., Staplin N., Brightling C., Ustianowski A., RECOVERY Collaborative Group (2020). Dexamethasone in Hospitalized Patients with COVID-19. N. Engl. J. Med..

[206] Abani O., Abbas A., Abbas F., Abbas M., Abbasi S., Abbass H., Abbott A., Abdallah N., Abdelaziz A., Abdelfattah M. (2021). Tocilizumab in patients admitted to hospital with COVID-19 (RECOVERY): A randomised, controlled, open-label, platform trial. Lancet.

[207] Auphan N., DiDonato J.A., Rosette C., Helmberg A., Karin M. (1995). Immunosuppression by glucocorticoids: Inhibition of NF-κB activity through induction of IκB synthesis. Science.

[208] Kandasamy M. (2021). NF-κB signalling as a pharmacological target in COVID-19: Potential roles for IKKβ inhibitors. Naunyn Schmiedebergs. Arch. Pharmacol..

[209] Cavalli G., de Luca G., Campochiaro C., Della-Torre E., Ripa M., Canetti D., Oltolini C., Castiglioni B., Din C.T., Boffini N. (2020). Interleukin-1 blockade with high-dose anakinra in patients with COVID-19, acute respiratory distress syndrome, and hyperinflammation: A retrospective cohort study. Lancet Rheumatol..

[210] Dimopoulos G., de Mast Q., Markou N., Theodorakopoulou M., Komnos A., Mouktaroudi M., Netea M.G., Spyridopoulos T., Verheggen R.J., Hoogerwerf J. (2020). Favorable anakinra responses in severe COVID-19 patients with secondary hemophagocytic lymphohistiocytosis. Cell Host Microbe.

[211] Huet T., Beaussier H., Voisin O., Jouveshomme S., Dauriat G., Lazareth I., Sacco E., Naccache J.-M., Bézie Y., Laplanche S. (2020). Anakinra for severe forms of COVID-19: A cohort study. Lancet Rheumatol..

[212] Navarro-Millán I., Sattui S.E., Lakhanpal A., Zisa D., Siegel C.H., Crow M.K. (2020). Use of anakinra to prevent mechanical ventilation in severe COVID-19: A case series. Arthritis Rheumatol..

[213] Pontali E., Volpi S., Antonucci G., Castellaneta M., Buzzi D., Tricerri F., Angelelli A., Caorsi R., Feasi M., Calautti F. (2020). Safety and efficacy of early high-dose IV anakinra in severe COVID-19 lung disease. J. Allergy Clin. Immunol..

[214] Shakoory B., Carcillo J.A., Chatham W.W., Amdur R.L., Zhao H., Dinarello C.A., Cron R.Q., Opal S.M. (2016). Interleukin-1 receptor blockade is associated with reduced mortality in sepsis patients with features of the macrophage activation syndrome: Re-analysis of a prior Phase III trial. Crit. Care Med..

[215] Guo Q., Wang Y., Xu D., Nossent J., Pavlos N.J., Xu J. (2018). Rheumatoid arthritis: Pathological mechanisms and modern pharmacologic therapies. Bone Res..

[216] Silman A.J., Pearson J.E. (2002). Epidemiology and genetics of rheumatoid arthritis. Arthritis Res..

[217] Asahara H., Asanuma M., Ogawa N., Nishibayashi S., Inoue H. (1995). High DNA-binding activity of transcription factor NF-kappa B in synovial membranes of patients with rheumatoid arthritis. Biochem. Mol. Biol. Int..

[218] Gilston V., Jones H.W., Soo C.C., Coumbe A., Blades S., Kaltschmidt C., Baeuerle P.A., Morris C.J., Blake D.R., Winyard P.G. (1997). NF-kappa B activation in human knee-joint synovial tissue during the early stage of joint inflammation. Biochem. Soc. Trans..

[219] Marok R., Winyard P., Coumbe A., Kus M., Gaffney K., Blades S., Mapp P., Morris C., Blake D., Kaltschmidt C. (1996). Activation of the transcription factor nuclear factor-κB in human inflamed synovial tissue. Arthritis Rheum. Off. J. Am. Coll. Rheumatol..

[220] Handel M.L., McMorrow L.B., Gravallese E.M. (1995). Nuclear factor-kappa B in rheumatoid synovium. Localization of p50 and p65. Arthritis Rheum..

[221] Miagkov A.V., Kovalenko D.V., Brown C.E., Didsbury J.R., Cogswell J.P., Stimpson S.A., Baldwin A.S., Makarov S.S. (1998). NF-kappaB activation provides the potential link between inflammation and hyperplasia in the arthritic joint. Proc. Natl. Acad. Sci. USA.

[222] Palombella V.J., Conner E.M., Fuseler J.W., Destree A., Davis J.M., Laroux F.S., Wolf R.E., Huang J., Brand S., Elliott P.J. (1998). Role of the proteasome and NF-kappaB in streptococcal cell wall-induced polyarthritis. Proc. Natl. Acad. Sci. USA.

[223] Tak P.P., Gerlag D.M., Aupperle K.R., van de Geest D.A., Overbeek M., Bennett B.L., Boyle D.L., Manning A.M., Firestein G.S. (2001). Inhibitor of nuclear factor kappaB kinase beta is a key regulator of synovial inflammation. Arthritis Rheum..

[224] Han Z., Boyle D.L., Manning A.M., Firestein G.S. (1998). AP-1 and NF-kappaB regulation in rheumatoid arthritis and murine collagen-induced arthritis. Autoimmunity.

[225] Davignon J.L., Hayder M., Baron M., Boyer J.F., Constantin A., Apparailly F., Poupot R., Cantagrel A. (2013). Targeting monocytes/macrophages in the treatment of rheumatoid arthritis. Rheumatology.

[226] Baum R., Gravallese E.M. (2016). Bone as a Target Organ in Rheumatic Disease: Impact on Osteoclasts and Osteoblasts. Clin. Rev. Allergy Immunol..

[227] Takayanagi H. (2007). Osteoimmunology: Shared mechanisms and crosstalk between the immune and bone systems. Nat. Rev. Immunol..

[228] Simmonds R.E., Foxwell B.M. (2008). Signalling, inflammation and arthritis: NF-kappaB and its relevance to arthritis and inflammation. Rheumatology.

[229] Evans H.G., Gullick N.J., Kelly S., Pitzalis C., Lord G.M., Kirkham B.W., Taams L.S. (2009). In vivo activated monocytes from the site of inflammation in humans specifically promote Th17 responses. Proc. Natl. Acad. Sci. USA.

[230] Sehnert B., Burkhardt H., Dübel S., Voll R.E. (2020). Cell-Type Targeted NF-kappaB Inhibition for the Treatment of Inflammatory Diseases. Cells.

[231] Tateiwa D., Yoshikawa H., Kaito T. (2019). Cartilage and Bone Destruction in Arthritis: Pathogenesis and Treatment Strategy: A Literature Review. Cells.

[232] Yap H.-Y., Tee S.Z.-Y., Wong M.M.-T., Chow S.-K., Peh S.-C., Teow S.-Y. (2018). Pathogenic Role of Immune Cells in Rheumatoid Arthritis: Implications in Clinical Treatment and Biomarker Development. Cells.

[233] Dong C. (2008). TH17 cells in development: An updated view of their molecular identity and genetic programming. Nat. Rev. Immunol..

[234] Sun S.C., Chang J.H., Jin J. (2013). Regulation of nuclear factor-κB in autoimmunity. Trends Immunol..

[235] Teng M.W., Bowman E.P., McElwee J.J., Smyth M.J., Casanova J.L., Cooper A.M., Cua D.J. (2015). IL-12 and IL-23 cytokines: From discovery to targeted therapies for immune-mediated inflammatory diseases. Nat. Med..

[236] Mackay F., Schneider P. (2009). Cracking the BAFF code. Nat. Rev. Immunol..

[237] Thompson N., Isenberg D.A., Jury E.C., Ciurtin C. (2016). Exploring BAFF: Its expression, receptors and contribution to the immunopathogenesis of Sjögren’s syndrome. Rheumatology.

[238] Wei F., Chang Y., Wei W. (2015). The role of BAFF in the progression of rheumatoid arthritis. Cytokine.

[239] Woo Y.J., Yoon B.Y., Jhun J.Y., Oh H.J., Min S.W., Cho M.L., Park S.H., Kim H.Y., Min J.K. (2011). Regulation of B cell activating factor (BAFF) receptor expression by NF-ΚB signaling in rheumatoid arthritis B cells. Exp. Mol. Med..

[240] Nejatbakhsh Samimi L., Farhadi E., Tahmasebi M.N., Jamshidi A., Vaziri A.S., Mahmoudi M. (2020). NF-κB signaling in rheumatoid arthritis with focus on fibroblast-like synoviocytes. Autoimmun. Highlights.

[241] Tas S.W., de Jong E.C., Hajji N., May M.J., Ghosh S., Vervoordeldonk M.J., Tak P.P. (2005). Selective inhibition of NF-κB in dendritic cells by the NEMO-binding domain peptide blocks maturation and prevents T cell proliferation and polarization. Eur. J. Immunol..

[242] Vomero M., Barbati C., Colasanti T., Perricone C., Novelli L., Ceccarelli F., Spinelli F.R., di Franco M., Conti F., Valesini G. (2018). Autophagy and rheumatoid arthritis: Current knowledges and future perspectives. Front. Immunol..

[243] Tas S.W., Vervoordeldonk M.J., Hajji N., Schuitemaker J.H., van der Sluijs K.F., May M.J., Ghosh S., Kapsenberg M.L., Tak P.P., de Jong E.C. (2007). Noncanonical NF-κB signaling in dendritic cells is required for indoleamine 2, 3-dioxygenase (IDO) induction and immune regulation. Blood J. Am. Soc. Hematol..

[244] Dhar A., Chawla M., Chattopadhyay S., Oswal N., Umar D., Gupta S., Bal V., Rath S., George A., Arimbasseri G.A. (2019). Role of NF-kappaB2-p100 in regulatory T cell homeostasis and activation. Sci. Rep..

[245] Murray S.E. (2013). Cell-intrinsic role for NF-kappa B-inducing kinase in peripheral maintenance but not thymic development of Foxp3+ regulatory T cells in mice. PLoS ONE.

[246] Gardam S., Sierro F., Basten A., Mackay F., Brink R. (2008). TRAF2 and TRAF3 Signal Adapters Act Cooperatively to Control the Maturation and Survival Signals Delivered to B Cells by the BAFF Receptor. Immunity.

[247] Weber A.N.R., Bittner Z., Liu X., Dang T.-M., Radsak M.P., Brunner C. (2017). Bruton’s Tyrosine Kinase: An Emerging Key Player in Innate Immunity. Front. Immunol..

[248] Ito M., Shichita T., Okada M., Komine R., Noguchi Y., Yoshimura A., Morita R. (2015). Bruton’s tyrosine kinase is essential for NLRP3 inflammasome activation and contributes to ischaemic brain injury. Nat. Commun..

[249] Sochorová K.R., Horváth R., Rozková D., Litzman J.I., Bartunková J.I., Sedivá A., Spísek R. (2006). Impaired Toll-like receptor 8–mediated IL-6 and TNF-α production in antigen-presenting cells from patients with X-linked agammaglobulinemia. Blood.

[250] Mukherjee S., Raje N., Schoonmaker J.A., Liu J.C., Hideshima T., Wein M.N., Jones D.C., Vallet S., Bouxsein M.L., Pozzi S. (2008). Pharmacologic targeting of a stem/progenitor population in vivo is associated with enhanced bone regeneration in mice. J. Clin. Investig..

[251] Garrett I., Chen D., Gutierrez G., Zhao M., Escobedo A., Rossini G., Harris S., Gallwitz W., Kim K., Hu S. (2003). Selective inhibitors of the osteoblast proteasome stimulate bone formation in vivo and in vitro. J. Clin. Investig..

[252] Giuliani N., Morandi F., Tagliaferri S., Lazzaretti M., Bonomini S., Crugnola M., Mancini C., Martella E., Ferrari L., Tabilio A. (2007). The proteasome inhibitor bortezomib affects osteoblast differentiation in vitro and in vivo in multiple myeloma patients. Blood J. Am. Soc. Hematol..

[253] Zangari M., Esseltine D., Lee C.-K., Barlogie B., Elice F., Burns M.J., Kang S.-H., Yaccoby S., Najarian K., Richardson P. (2005). Response to bortezomib is associated to osteoblastic activation in patients with multiple myeloma. Br. J. Haematol..

[254] Hirano T., Murakami M. (2020). COVID-19: A New Virus, but a Familiar Receptor and Cytokine Release Syndrome. Immunity.

[255] Huang C., Wang Y., Li X., Ren L., Zhao J., Hu Y., Zhang L., Fan G., Xu J., Gu X. (2020). Clinical features of patients infected with 2019 novel coronavirus in Wuhan, China. Lancet.

[256] Costela-Ruiz V.J., Illescas-Montes R., Puerta-Puerta J.M., Ruiz C., Melguizo-Rodríguez L. (2020). SARS-CoV-2 infection: The role of cytokines in COVID-19 disease. Cytokine Growth Factor Rev..

[257] Gao Y.M., Xu G., Wang B., Liu B.C. (2020). Cytokine storm syndrome in coronavirus disease 2019: A narrative review. J. Intern. Med..

[258] Hariharan A., Hakeem A.R., Radhakrishnan S., Reddy M.S., Rela M. (2020). The Role and Therapeutic Potential of NF-kappa-B Pathway in Severe COVID-19 Patients. Inflammopharmacology.

[259] Liao Q.J., Ye L.B., Timani K.A., Zeng Y.C., She Y.L., Ye L., Wu Z.H. (2005). Activation of NF-kappaB by the full-length nucleocapsid protein of the SARS coronavirus. Acta Biochim. Biophys. Sin..

[260] Wu Y., Ma L., Cai S., Zhuang Z., Zhao Z., Jin S., Xie W., Zhou L., Zhang L., Zhao J. (2021). RNA-induced liquid phase separation of SARS-CoV-2 nucleocapsid protein facilitates NF-κB hyper-activation and inflammation. Signal Transduct. Target. Ther..

[261] Dosch S.F., Mahajan S.D., Collins A.R. (2009). SARS coronavirus spike protein-induced innate immune response occurs via activation of the NF-kappaB pathway in human monocyte macrophages in vitro. Virus. Res..

[262] DeDiego M.L., Nieto-Torres J.L., Regla-Nava J.A., Jimenez-Guardeño J.M., Fernandez-Delgado R., Fett C., Castaño-Rodriguez C., Perlman S., Enjuanes L. (2014). Inhibition of NF-κB-mediated inflammation in severe acute respiratory syndrome coronavirus-infected mice increases survival. J. Virol..

[263] de Wit E., van Doremalen N., Falzarano D., Munster V.J. (2016). SARS and MERS: Recent insights into emerging coronaviruses. Nat. Rev. Microbiol..

[264] Totura A.L., Whitmore A., Agnihothram S., Schäfer A., Katze M.G., Heise M.T., Baric R.S. (2015). Toll-Like Receptor 3 Signaling via TRIF Contributes to a Protective Innate Immune Response to Severe Acute Respiratory Syndrome Coronavirus Infection. mBio.

[265] Khan S., Shafiei M.S., Longoria C., Schoggins J.W., Savani R.C., Zaki H. (2021). SARS-CoV-2 spike protein induces inflammation via TLR2-dependent activation of the NF-κB pathway. Elife.

[266] Singh A.K., Majumdar S., Singh R., Misra A. (2020). Role of corticosteroid in the management of COVID-19: A systemic review and a Clinician’s perspective. Diabetes Metab. Syndr. Clin. Res. Rev..

[267] Cavalli G., Farina N., Campochiaro C., de Luca G., Della-Torre E., Tomelleri A., Dagna L. (2020). Repurposing of Biologic and Targeted Synthetic Anti-Rheumatic Drugs in COVID-19 and Hyper-Inflammation: A Comprehensive Review of Available and Emerging Evidence at the Peak of the Pandemic. Front. Pharmacol..

[268] Weisberg E., Parent A., Yang P.L., Sattler M., Liu Q., Liu Q., Wang J., Meng C., Buhrlage S.J., Gray N. (2020). Repurposing of Kinase Inhibitors for Treatment of COVID-19. Pharm. Res..

[269] Bahadoram M., Keikhaei B., Saeedi-Boroujeni A., Mahmoudian-Sani M.-R. (2021). Chloroquine/hydroxychloroquine: An inflammasome inhibitor in severe COVID-19?. Naunyn-Schmiedeberg’s Arch. Pharmacol..

[270] Bai L., Li J., Li H., Song J., Zhou Y., Lu R., Liu B., Pang Y., Zhang P., Chen J. (2019). Renoprotective effects of artemisinin and hydroxychloroquine combination therapy on IgA nephropathy via suppressing NF-κB signaling and NLRP3 inflammasome activation by exosomes in rats. Biochem. Pharmacol..

[271] Tang T.-T., Lv L.-L., Pan M.-M., Wen Y., Wang B., Li Z.-L., Wu M., Wang F.-M., Crowley S.D., Liu B.-C. (2018). Hydroxychloroquine attenuates renal ischemia/reperfusion injury by inhibiting cathepsin mediated NLRP3 inflammasome activation. Cell Death Dis..

[272] Liu J., Cao R., Xu M., Wang X., Zhang H., Hu H., Li Y., Hu Z., Zhong W., Wang M. (2020). Hydroxychloroquine, a less toxic derivative of chloroquine, is effective in inhibiting SARS-CoV-2 infection in vitro. Cell Discov..

[273] Yao X., Ye F., Zhang M., Cui C., Huang B., Niu P., Liu X., Zhao L., Dong E., Song C. (2020). In Vitro Antiviral Activity and Projection of Optimized Dosing Design of Hydroxychloroquine for the Treatment of Severe Acute Respiratory Syndrome Coronavirus 2 (SARS-CoV-2). Clin. Infect. Dis..

[274] Gautret P., Lagier J.C., Parola P., Hoang V.T., Meddeb L., Mailhe M., Doudier B., Courjon J., Giordanengo V., Vieira V.E. (2020). Hydroxychloroquine and azithromycin as a treatment of COVID-19: Results of an open-label non-randomized clinical trial. Int. J. Antimicrob. Agents.

[275] Lenzer J. (2020). COVID-19: US gives emergency approval to hydroxychloroquine despite lack of evidence. BMJ.

[276] Mahase E. (2020). Hydroxychloroquine for Covid-19: The end of the line?. BMJ.

[277] Cavalcanti A.B., Zampieri F.G., Rosa R.G., Azevedo L.C.P., Veiga V.C., Avezum A., Damiani L.P., Marcadenti A., Kawano-Dourado L., Lisboa T. (2020). Hydroxychloroquine with or without Azithromycin in Mild-to-Moderate COVID-19. N. Engl. J. Med..

[278] Consortium W.S.T. (2021). Repurposed antiviral drugs for COVID-19—Interim WHO SOLIDARITY trial results. N. Engl. J. Med..

[279] Group R.C. (2020). Effect of hydroxychloroquine in hospitalized patients with COVID-19. N. Engl. J. Med..

[280] Jorge A. (2021). Hydroxychloroquine in the prevention of COVID-19 mortality. Lancet Rheumatol..

[281] Magagnoli J., Narendran S., Pereira F., Cummings T.H., Hardin J.W., Sutton S.S., Ambati J. (2020). Outcomes of Hydroxychloroquine Usage in United States Veterans Hospitalized with COVID-19. Med.

[282] Mahévas M., Tran V.-T., Roumier M., Chabrol A., Paule R., Guillaud C., Fois E., Lepeule R., Szwebel T.-A., Lescure F.-X. (2020). Clinical efficacy of hydroxychloroquine in patients with COVID-19 pneumonia who require oxygen: Observational comparative study using routine care data. BMJ.

[283] Mitjà O., Corbacho-Monné M., Ubals M., Tebé C., Peñafiel J., Tobias A., Ballana E., Alemany A., Riera-Martí N., Pérez C.A. (2020). Hydroxychloroquine for Early Treatment of Adults With Mild Coronavirus Disease 2019: A Randomized, Controlled Trial. Clin. Infect. Dis..

[284] Mullard A. (2021). RECOVERY 1 year on: A rare success in the COVID-19 clinical trial landscape. Nat. Rev. Drug Discov..

[285] Pathak D.S.K., Salunke D.A.A., Thivari D.P., Pandey A., Nandy D.K., Harish V.K.R.D., Pandey D.S., Chawla D.J., Mujawar D.J., Dhanwate D.A. (2020). No benefit of hydroxychloroquine in COVID-19: Results of Systematic Review and Meta-Analysis of Randomized Controlled Trials. Diabetes Metab. Syndr..

[286] Rosenberg E.S., Dufort E.M., Udo T., Wilberschied L.A., Kumar J., Tesoriero J., Weinberg P., Kirkwood J., Muse A., DeHovitz J. (2020). Association of treatment with hydroxychloroquine or azithromycin with in-hospital mortality in patients with COVID-19 in New York State. JAMA.

[287] Skipper C.P., Pastick K.A., Engen N.W., Bangdiwala A.S., Abassi M., Lofgren S.M., Williams D.A., Okafor E.C., Pullen M.F., Nicol M.R. (2020). Hydroxychloroquine in Nonhospitalized Adults With Early COVID-19: A Randomized Trial. Ann. Intern. Med..

[288] Chatre C., Roubille F., Vernhet H., Jorgensen C., Pers Y.M. (2018). Cardiac Complications Attributed to Chloroquine and Hydroxychloroquine: A Systematic Review of the Literature. Drug Saf..

[289] Tleyjeh I.M., Kashour Z., AlDosary O., Riaz M., Tlayjeh H., Garbati M.A., Tleyjeh R., Al-Mallah M.H., Sohail M.R., Gerberi D. (2021). Cardiac Toxicity of Chloroquine or Hydroxychloroquine in Patients With COVID-19: A Systematic Review and Meta-regression Analysis. Mayo Clin. Proc. Innov. Qual. Outcomes.

[290] Cohen I.V., Makunts T., Moumedjian T., Issa M.A., Abagyan R. (2020). Cardiac adverse events associated with chloroquine and hydroxychloroquine exposure in 20 years of drug safety surveillance reports. Sci. Rep..

[291] Stevenson A., Kirresh A., Conway S., White L., Ahmad M., Little C. (2020). Hydroxychloroquine use in COVID-19: Is the risk of cardiovascular toxicity justified?. Open Heart.

[292] FDA (2020). Coronavirus (COVID-19) Update: FDA Revokes Emergency Use Authorization for Chloroquine and Hydroxychloroquine. https://www.fda.gov/news-events/press-announcements/coronavirus-covid-19-update-fda-revokes-emergency-use-authorization-chloroquine-and.

[293] Perkins N.D. (2006). Post-translational modifications regulating the activity and function of the nuclear factor kappa B pathway. Oncogene.

[294] Hoffmann A., Natoli G., Ghosh G. (2006). Transcriptional regulation via the NF-κB signaling module. Oncogene.

[295] Begalli F., Bennett J., Capece D., Verzella D., D’Andrea D., Tornatore L., Franzoso G. (2017). Unlocking the NF-κB Conundrum: Embracing Complexity to Achieve Specificity. Biomedicines.

[296] Bennett J., Capece D., Begalli F., Verzella D., D’Andrea D., Tornatore L., Franzoso G. (2018). NF-κB in the crosshairs: Rethinking an old riddle. Int. J. Biochem. Cell Biol..

[297] Greten F.R., Arkan M.C., Bollrath J., Hsu L.C., Goode J., Miething C., Göktuna S.I., Neuenhahn M., Fierer J., Paxian S. (2007). NF-kappaB is a negative regulator of IL-1beta secretion as revealed by genetic and pharmacological inhibition of IKKbeta. Cell.

[298] Hsu L.-C., Enzler T., Seita J., Timmer A.M., Lee C.-Y., Lai T.-Y., Yu G.-Y., Lai L.-C., Temkin V., Sinzig U. (2011). IL-1β-driven neutrophilia preserves antibacterial defense in the absence of the kinase IKKβ. Nat. Immunol..

[299] Sehnert B., Burkhardt H., Wessels J.T., Schröder A., May M.J., Vestweber D., Zwerina J., Warnatz K., Nimmerjahn F., Schett G. (2013). NF-κB inhibitor targeted to activated endothelium demonstrates a critical role of endothelial NF-κB in immune-mediated diseases. Proc. Natl. Acad. Sci. USA.

